# Hyperactivity of Rac1-GTPase pathway impairs neuritogenesis of cortical neurons by altering actin dynamics

**DOI:** 10.1038/s41598-018-25354-3

**Published:** 2018-05-08

**Authors:** Valentina Zamboni, Maria Armentano, Gaia Berto, Elisa Ciraolo, Alessandra Ghigo, Donatella Garzotto, Alessandro Umbach, Ferdinando DiCunto, Elena Parmigiani, Marina Boido, Alessandro Vercelli, Nadia El-Assawy, Alessandro Mauro, Lorenzo Priano, Luisa Ponzoni, Luca Murru, Maria Passafaro, Emilio Hirsch, Giorgio R. Merlo

**Affiliations:** 1 Department of Molecular Biotechnology and Health Sciences, University of Torino, Turin, Italy; 2 Neuroscience Institute –Cavalieri Ottolenghi, Orbassano (Torino), Turin, Italy; 3 Department of Neurosciences, University of Torino & Division Neurology and Neurorehabilitation, S. Giuseppe Hospital, IRCCS Istituto Auxologico Italiano, Piancavallo (VB), Turin, Italy; 4 Fondazione Umberto Veronesi, Milan, Italy; 5 Neuroscience Institute, Consiglio Nazionale Ricerche, Milan, Italy

## Abstract

The small-GTPase Rac1 is a key molecular regulator linking extracellular signals to actin cytoskeleton dynamics. Loss-of-function mutations in RAC1 and other genes of the Rac signaling pathway have been implicated in the pathogenesis of Intellectual Disability (ID). The Rac1 activity is negatively controlled by GAP proteins, however the effect of Rac1 hyperactivity on neuronal networking *in vivo* has been poorly studied. ArhGAP15 is a Rac-specific negative regulator, expressed in the main subtypes of pyramidal cortical neurons. In the absence of *ArhGAP15*, cortical pyramidal neurons show defective neuritogenesis, delayed axonal elongation, reduced dendritic branching, both *in vitro* and *in vivo*. These phenotypes are associated with altered actin dynamics at the growth cone due to increased activity of the PAK-LIMK pathway and hyperphosphorylation of ADF/cofilin. These results can be explained by shootin1 hypo-phosphorylation and uncoupling with the adhesion system. Functionally, *ArhGAP15*^−/−^ mice exhibit decreased synaptic density, altered electroencephalographic rhythms and cognitive deficits. These data suggest that both hypo- and hyperactivation of the Rac pathway due to mutations in Rac1 regulators can result in conditions of ID, and that a tight regulation of Rac1 activity is required to attain the full complexity of the cortical networks.

## Introduction

Intellectual Disability (ID) is a neurodevelopmental disorder characterized by significant impairments in both intellectual functioning and in adaptive skills. ID patients show defects in network connectivity and altered excitation/inhibition balance of cerebral cortex and hippocampus, and these alterations may result in abnormal information processing^1^.

Mutations of *RAC1* and of genes involved in the Rac-GTPase pathway such as *PAK3*, *ARHGEF9* and *ARHGEF6* (also known as α*PIX*) have been identified in patients with ID^1–5^. Collectively, the genetic variants identified in ID patients are all of the loss-of-function type, implying that a hypoactive main Rac pathway is the pathogenic cause of these conditions^1,2^, although the identification of the endophenotype underlying ID is far from being clarified.

Rac-GTPases are key molecular switches that link extracellular signals to actin cytoskeleton dynamics. They cycle between an inactive GDP-bound form and an active GTP-bound state, a binary switch that is tightly regulated by several guanine nucleotide exchange factors (GEFs), by GTPase-activating proteins (GAPs), and by guanine nucleotide dissociation inhibitors (GDIs)^6^. Over the past several years, it has become clear that Rac-GTPases and their regulators control the dynamics of actin cytoskeleton rearrangements, inducing stabilization and destabilization of the actin filament network, needed to transduce extracellular cues into vectorial flow of information^7,8^.

Rac1 acts by controlling actin dynamics at the growth cone and leading edge on immature neurons, through actin filament polymerization/disassembly, acto-myosin contractility and engagement of intracellular adhesion and anchoring mechanisms via regulation of actin-binding proteins (ABP)^9^.

A large body of experimental evidence has documented the role of aberrant function of components of the Rac1 signaling pathway in neurodevelopmental disorders, such as ID, however our current knowledge on Rac-GTPases function is obtained *in vitro* by overexpressing the constitutively active or dominant-negative Rac1 mutant proteins, which may have significant drawbacks^10,11^. On the other hand, *in vivo* studies have been focused on the analysis of mice with loss-of-function mutations of *Rac1/Rac3*, pointing out specific functions of these GTPases during development of both the excitatory and the inhibitory networks. For instance in the mouse, the *synapsin1-cre*-mediated conditional deletion of *Rac1* in neurons (*Rac-N*), also in combination with *Rac3* deletion, leads to migration, differentiation and connectivity defects^10,12–15^. A complete knowledge of Rac1 regulation during neuronal network formation is still lacking. For instance, little is known about the role of the several neuronal-specific positive and negative regulators of Rac1, or the effect of Rac hyperactivity on neuronal networking, *in vivo*. Notably, the loss of function of the Rho-specific GAP *oligophrenin-1* in humans is responsible for X-linked syndromic ID^16^.

ArhGAP15 is a Rac-specific GAP that negatively regulates Rac1/3 activity^17,18^. In human, loss of *ArhGAP15* has been associated to cognitive disorders, in the Mowat-Wilson disease, characterized by severe neurological and cognitive deficits, severe ID and speech impairment, and in most cases epilepsy^19–22^. In the most severe variants of the Mowat-Wilson syndrome, the loss of *ArhGAP15* accompanies the deletion of *Zeb2*, the known disease gene. Thus the absence of *ArhGAP15* in humans might contribute to severe the pathological condition. *In vitro*, the over-expression of *ArhGAP15* results in increased actin stress fibers and cell contraction^17,23,24^, suggesting that properly regulated inhibition of the Rac1 pathway is essential for cytoskeletal reorganization.

In this work we addressed the question of whether the loss of a Rac-specific negative regulator may affect neuronal development and axon outgrowth during development of the nervous system, through the hyperactivation of the Rac1 downstream pathway. To do this, we analyzed the molecular and cellular consequences of the genetic deletion of *ArhGAP15* in mice^18,23^. We find that the absence of *ArhGAP15* results in defective neuritogenesis, dendritic complexity and axon elongation of cortical pyramidal neurons, and reduced synaptic density. These phenotypes are associated with altered actin dynamics at the growth cone due to increased activity of the PAK-LIMK pathway and hyperphosphorylation of ADF/cofilin. Functionally, *ArhGAP15*^−/−^ mice exhibit altered electroencephalographic rhythms and cognitive deficits.

## Results

### Expression of *ArhGAP15* in the postnatal mouse brain

**Figure 1:**
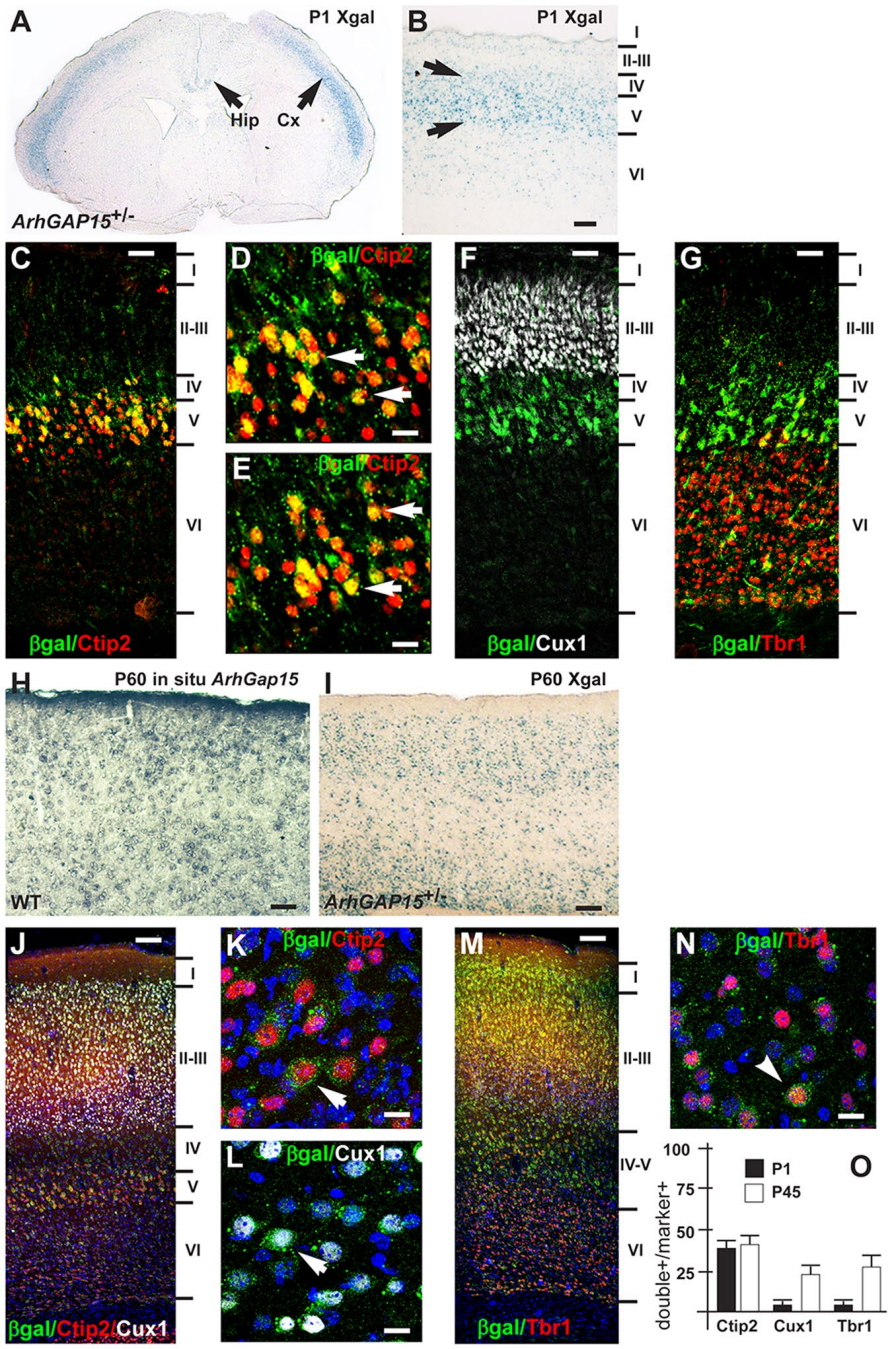
Expression of *ArhGAP15* in early postnatal and adult mouse brain. (**A**,**B**) Xgal staining of coronal sections of the *ArhGAP15*^+/−^ brain, at postnatal day 1 (P1). In (**A**), low magnification; in (**B**), a higher magnification of the somatosensory area. Cortical layers are indicated on the right. Signal is detected mainly in layer V. Scale bar in B = 60 μm. (**C**–**G**) Immunostaining of the somatosensory cortex of *ArhGAP15*^+/−^ brains, at P1, with anti-βgal (e.g. ArhGAP15, green cytoplasmic stain) and either Ctip2 (**C**,**D**,**E**, red nuclear fluorescence), Cux1 (**F**, white nuclear stain) or Tbr1 (**G**, red nuclear stain) pyramidal markers. In (**D**) and (**E**), higher magnification from the image in (**C**). Double-stained neurons were observed only with Ctip2 (**C**–**E**), indicated with white arrows. Scale bars in C,F,G = 50 μm, bar in D,E = 20 μm. (**H**) RNA::RNA *in situ* hybridization to detect the *ArhGAP15* mRNA in coronal sections of the somatosensory cortex of young adult (P60) WT animals. Scale bar = 50 μm. (**I**) Xgal staining of coronal sections of the somatosensory cortex from *ArhGAP15*^+/−^ P60 animals. Scale bar = 60 μm. (**J–N**) Characterization of βgal-expressing (green cytoplasmic staining) pyramidal neurons in *ArhGAP15*^+/−^ brains at the age P45, by double immunostaining with the layer-specific markers Ctip2 (red nuclear staining), Cux1 (white nuclear staining) and Tbr1 (red nuclear staining). (**J** and **M**) show low magnification images, with cortical layers indicated on the right. K,L and N show higher magnifications from the images on the left. Double-positive cells are indicated with white arrows. (**O**) Percentages of Ctip2-, Cux1- and Tbr1-positive pyramidal neurons that stain double-positive for βgal, at P1 (solid bars) and P45 (open bars). Quantification was done by section. Scale bars in J,M = 50 μm, bar in K,L,N = 10 μm. Data are presented as mean ± SEM.

We previously showed that in the early postnatal brain, ArhGAP15 is expressed in the olfactory bulbs, cortex and hippocampus^18^. Xgal staining of newborn (P1) *ArhGAP15*^+/−^ brains showed abundant βgal-expressing cells in the somatosensory and somatomotor cortical areas (Fig. 1A,B). We characterized the expression in pyramidal neurons by double immunostaining of *ArhGAP15*^+/−^ brains at the age P1 with anti-βgal (detecting ArhGAP15-expressing cells) and either Ctip2, Cux1 or Tbr1 (markers, respectively, of pyramidal neurons in layer V, layers II-III and layer VI). The use of anti-βgal is justified by the fact that existing antibodies directed against ArhGAP15 also detect unspecific signal, and cannot be reliably used. βgal/ArhGAP15 was detected nearly exclusively in Ctip2+ neurons in layer V, while it was not detected in Cux1+ and Tbr1+ neurons in other layers (Fig. 1C–G). Quantification of the double-stained neurons indicated that only a fraction (40% ± 3.84%) of the Ctip2+ neurons is βgal+ (Fig. 1O).

In young adults (P60) we carried out RNA::RNA *in situ* hybridization on coronal sections of WT brains, to detect the *ArhGAP15* mRNA; expression was observed in the olfactory bulbs, cortex and hippocampus (Fig. 1H). Similarly, *in situ* hybridization with a probe detecting the *lacZ* mRNA on *ArhGAP15*^+/−^ brains showed an identical expression pattern. We then carried out Xgal staining of coronal sections of P60 *ArhGAP15*^+/−^ brains and again we observed expression of βgal in all regions of the cerebral cortex as well as the hippocampus (Fig. 1I). Finally, we carried out Xgal staining and the *lacZ* RNA::RNA *in situ* hybridization to compare *ArhGAP15*^+/−^ and *ArhGAP15*^−/−^ brains, and observed no difference between the two genotypes (Supplementary Fig. S1A,B).

To examine the expression of ArhGAP15 in young adult pyramidal neurons in more details, we carried out double immunostaining on *ArhGAP15*^+/−^ brains at the age P45, to detect βgal and either Ctip2, Cux1 and Tbr1, markers of layer-specific and target-specific subclasses of cortical pyramidal neurons. In the adult *ArhGAP15*^+/−^ brain βgal/ArhGAP15 was detected in a fraction of all pyramidal neuron subtypes (Ctip2/βgal 43% ± 4.80%; Cux1/βgal24% ± 2.38%; Tbr1/βgal 28% ± 7.45%) (Fig. 1J–O). In summary ArhGAP15 is expressed postmitotically, in a time-dependent manner, in a fraction of most subtypes of cortical pyramidal neurons, the first ones being Ctip2+ neurons in layer V.

### Cortical organization is normal in *ArhGAP15*^−/−^ mice

Xgal staining and *lacZ* RNA::RNA *in situ* hybridization showed no evident difference between normal and *ArhGAP15*^−/−^ brains in terms of distribution of the reporter-expressing cells (Supplementary Fig. S1A,B), indicating that the *ArhGAP15/lacZ* expressing cells are not lost or grossly mislocalized in the absence of *ArhGAP15*. In addition, immunostaining of P1 brain sections for Ctip2 showed no difference in the neuron number and position (Supplementary Fig. S1C,D). Furthermore, staining of coronal sections of the early post-natal or adult animals with TUNEL for the detection of apoptotic cells revealed no significant change in the number and position of TUNEL+ cells, thus we exclude a loss of specific cell populations.

**Figure 2:**
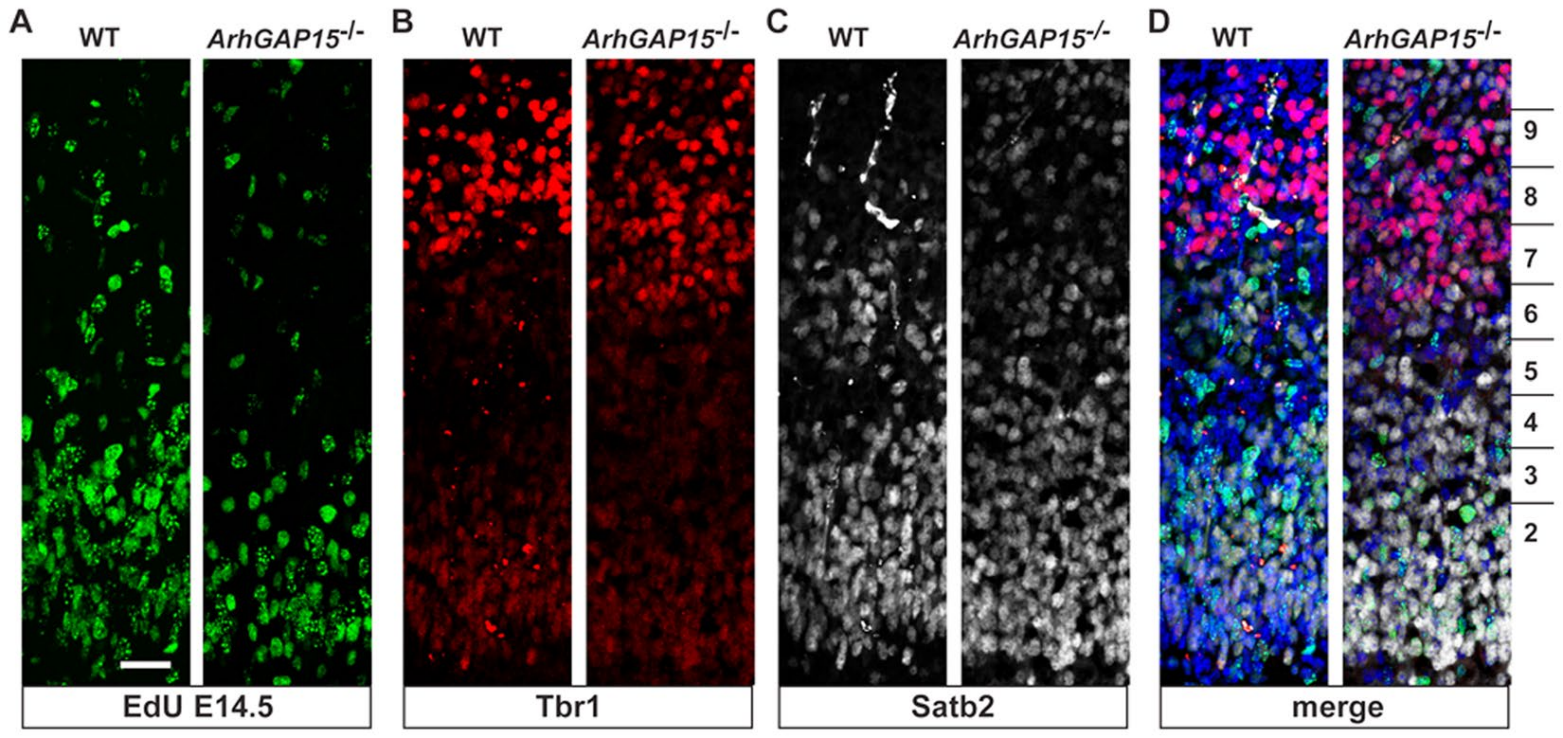
Genesis and organization of cortical pyramidal neurons in *ArhGAP15*^−/−^ mice. (**A**) Representative micrographs of EdU tracing of cortical neurons in WT (left) and *ArhGAP15*^−/−^ (right) cortices, examined at birth. Embryos were injected with EdU at the age E14.5. No difference in the number and position of EdU+ neurons is detected. (**B**–**D**) Representative micrographs of immunostaining of WT and *ArhGAP15*^−/−^ cortices, at birth, for Tbr1 (**B**) and for Satb2 (**C**). The merged image (DAPI, EdU+, Tbr2+, Satb2+) is shown in panel D. The subdivision in 10 BINs is reported in panel D. No differences in the number and position of the stained neurons were detected. Scale bar in A = 20 μm.

To better evaluate the genesis and cyto-architecture of cortical pyramidal neurons in the absence of *ArhGAP15*, we pulse-labeled proliferating neuroblasts with a single injection of EdU at E14.5 and then determined the number and distribution of EdU+, Satb2+ and Tbr1+ neurons detected at birth. Satb2 is a postmitotic determinant for upper-layer cortical neurons^25^, while Tbr1 is preferentially expressed in layer VI neurons^26^. We observed no difference in either the laminar organization of Satb2 or Tbr1 pyramidal neurons or in the number/position of EdU-labeled neurons between the two genotypes (Fig. 2). We conclude that, at birth, in the absence of *ArhGAP15* pyramidal neurons are normally generated and positioned.

### Loss of *ArhGAP15* affects neuronal morphology and neuritogenesis *in vitro* and *in vivo*

**Figure 3:**
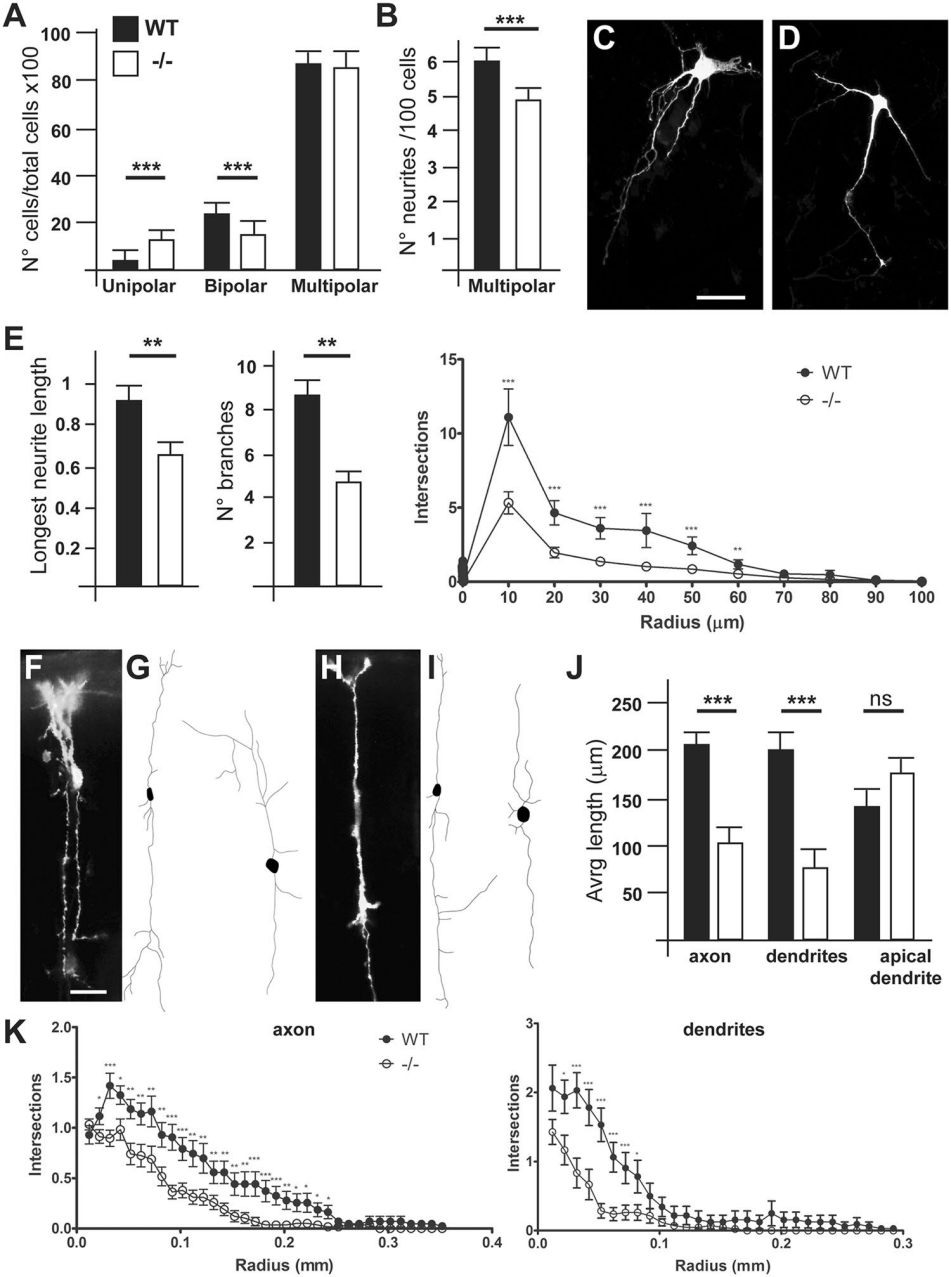
Loss of *ArhGAP15* affects neuronal morphology and neuritogenesis of cortical pyramidal neurons. (**A**) Relative proportion of unipolar, bipolar and multipolar neurons in primary cultures obtained from WT (solid bars) or *ArhGAP15*^−/−^ (open bars) embryonic cortices. Cells were electroporated with a PGK-GFP vector prior to plating, and examined after 3 DIV. In the absence of *ArhGAP15*, the number of bipolar neurons is significantly decreased, while the number of unipolar neurons is increased. Results are expressed as percentage over the total number of cells counted (minimum 150). (**B**) Multipolar neurons were further scored for the number of neurites (on the right), and in the absence of *ArhGAP15* neurons show a significantly decreased number of average neurites. (**C**,**D**) Representative micrographs of cortical neurons in primary culture from WT (left) or *ArhGAP15*^*−/−*^ (right) animals. Cells were transfected with a PGK-GFP vector prior to plating, and examined after 3 DIV. Only cells with a pyramidal morphology were considered (>90% of all neurons). Scale bar in C = 20 μm. (**E**) Quantification of the length of the longest neurite, of the number of branches (secondary neurites) and of the overall complexity of arborization (Sholl analysis) in neurons from WT (solid bars) and *ArhGAP15*^−/−^ (open bars) cortices. (**F**,**H**) Representative micrographs of DiI labelled cortical pyramidal neurons from WT (**F**) and *ArhGAP15*^−/−^ (**H**) animals at P2. (**G**,**I**) Examples of reconstructed DiI labelled cortical pyramidal neurons from WT (**G**) and *ArhGAP15*^−/−^ (**I**) animals at P2. (**J**) Quantification of the length of the axon, of the dendrites and of the apical dendrites in DiI labelled cortical pyramidal neurons from WT (solid bars) and *ArhGAP15*^−/−^ (open bars) animals at P2. (**K**) Sholl analysis of the axon (left) and dendritic complexity (right) revealed a significant reduction in the arborization of cortical pyramidal neurons from *ArhGAP15*^−/−^ animals. Data are presented as mean ± SEM. **P ≤ 0.01, ***P ≤ 0.001 (Student’s t-test).

The small-GTPase Rac1 is critical for the acquisition of neuronal morphology and for neuritogenesis during development^27^. To determine whether loss of *ArhGAP15*, hence hyperactive Rac1/Rac3, affects neuronal morphology, neurite elongation, branching and overall neurite complexity, we examined primary cultures of neurons dissociated from cortices of WT and *ArhGAP15*^−/−^ embryos, at the age E15.5, electroporated with a *Green Fluorescent Protein* (*GFP*) expressing vector and maintained 3 DIV (Supplementary Fig. S2A). First, we categorized neurons on the basis of the number of main neurites, and classified them as unipolar (having one visible neurite), bipolar (two neurites) and multipolar (>two neurites) (Supplementary Fig. S2B–D). We found that the fraction of unipolar cells was significantly increased (WT 5% vs. KO 12%) in cultures of *ArhGAP15*^−/−^ cortices, while the fraction of bipolar cells was diminished (WT 21% vs. KO 14%) (Fig. 3A). The fraction of multipolar neurons was unchanged in the absence of *ArhGAP15*^−/−^, however the number of their neurites is reduced in *ArhGAP15*^−/−^ neurons, compared to WT (WT 6 ± 0.18 vs. KO 5 ± 0.23) (Fig. 3B). These data indicate that *ArhGAP15* is required by immature neurons to achieve a more elaborated morphology, and in the absence of *ArhGAP15* the formation of the first extension might be delayed.

We then examined neurite length and complexity of pyramidal-looking neurons (which represent >90% of cultured neurons). *ArhGAP15*^−/−^ neurons displayed a reduced length of the main neurite, a reduced number of secondary neurites, and reduced intersection distribution and number of intersections (respectively, p = 2.3 10^−4^; 9.4 10^−7^ and 4.5 10^−11^) (Fig. 3C–E). Moreover, Sholl analysis revealed that the *ArhGAP15*^−/−^ pyramidal neurons display nearly one-third of the number of neurites stemming from the soma and a reduced number of branches crossing more distal concentric circles centered at the cell body, compared to the WT (Fig. 3E). These data indicate that in the absence of *ArhGAP15* neurons possess a simpler morphology and reduced efficiency of neuritogenesis and branching, *in vitro*.

To further investigate the axonal elongation and the dendrite branching, we evaluated the neuronal morphology and neurite length *in vivo*. We implanted crystals of DiI in the corpus callosum of sections of P2 brains from WT (N = 10 from 5 brains) and *ArhGAP15*^−/−^ (N = 10 from 5 brains). After 7 days of diffusion time, we examined the dendritic complexity of DiI-labelled callosal neurons (Fig. 3F,H) using the Neurolucida software (Fig. 3G,I). In the *ArhGAP15*^*−/−*^ brains we detected a reduced length of axons (WT 0.22 mm ± 0.03 vs. KO 0.11 mm ± 0.01) and dendrites (WT 0.22 mm ± 0.03 vs. KO 0.08 mm ± 0.02) (Fig. 3J). Moreover Sholl analysis indicates that *ArhGAP15*^−/−^ callosal neurons display a less complex neuronal arbor (Fig. 3K). These data indicate that ArhGAP15 is required by neurons to attain a mature morphology and a complex dendritic and axonal arborization.

### Loss of *ArhGAP15* reduces axonal elongation of callosal axons *in vivo*

**Figure 4:**
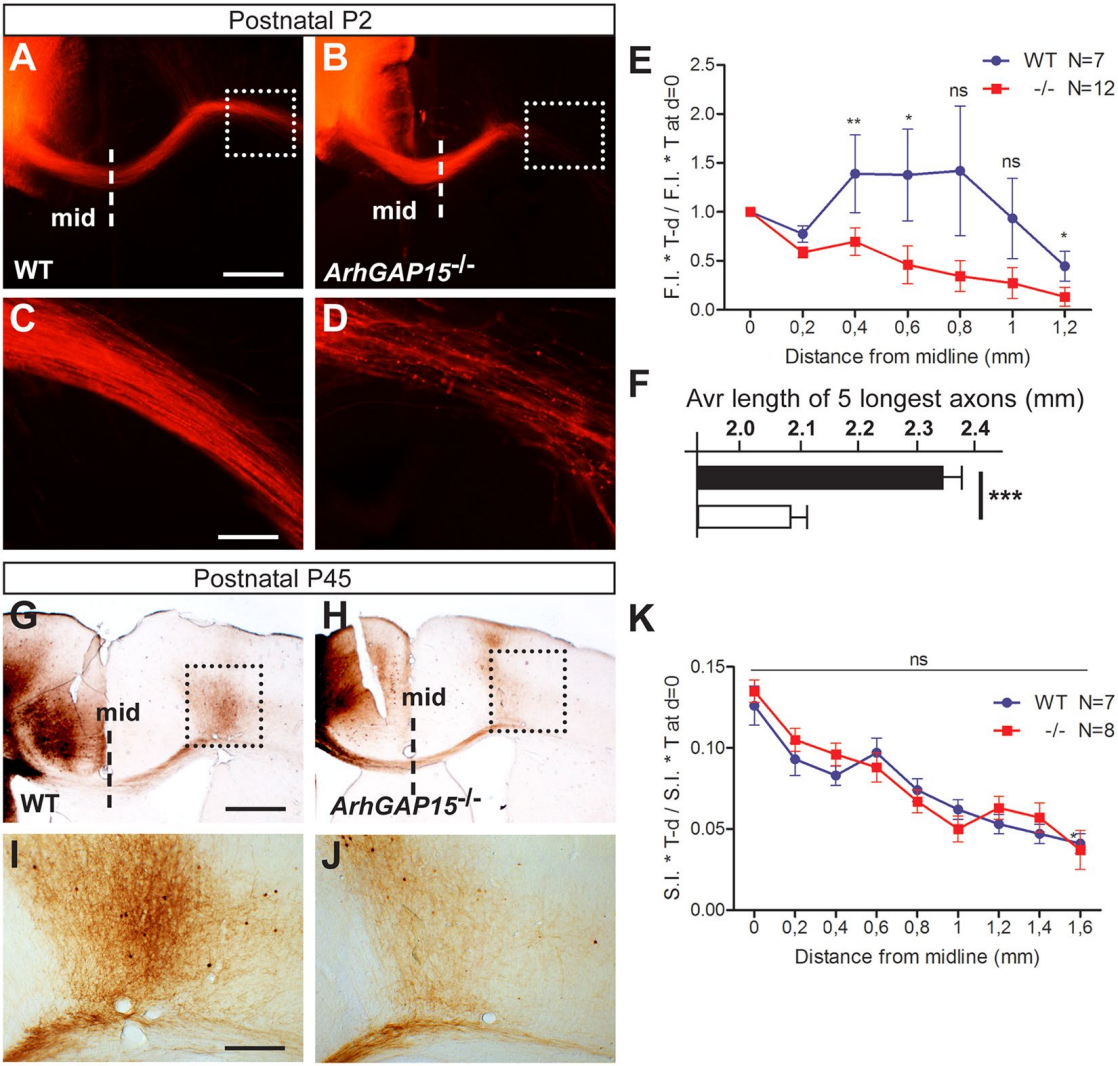
Loss of *ArhGAP15* affects the length and targeting of pyramidal callosal commissural axons *in vivo*. (**A**–**D**) Representative images of DiI-labeled callosal projection of WT (**A**,**C**) and *ArhGAP15*^*−/−*^ (**B**,**D**) P2 brains, for tracing analyses. Low magnification images are shown on the top, while the box inserts indicate the area shown at a higher magnification, on the bottom. (**E**) Quantification of the F.I. *thickness of the callosal bundle, normalized against the F.I. *thickness at the midline (indicated in panels A and B with dashed lines), as a function of the distance from the midline. 7 and 12 sections were examined for, respectively, the WT and *ArhGAP15* mutant genotypes, from at least 3 independent brains in both cases. (**F**) Average length from the midline, in mm, of the 5 longest DiI-labelled callosal axons, in WT (solid bars, N = 7) or *ArhGAP15*^*−/−*^ (open bars, N = 12) brain slices. (**G**–**J**) Representative images of BDA-labeled callosal projections of WT (**G**,**I**) and *ArhGAP15*^*−/−*^ (**H**,**J**) P45 brains, for tracing analyses. Images on the top are at low magnification, to show the injection site, the callosal projections, and the midline (reported in panels G and H with dashed lines). Images on the bottom are higher magnification of the inset indicated in the images on the top. Scale bars in A and G = 200 μm, bars in C and I = 20 μm. (**K**) Quantification of axonal length, as in panel E except that F.I. was substituted with S.I. (staining intensity). No difference was observed at this age. 7 and 8 comparable sections of the WT and *ArhGAP15*^−/−^ animals were examined, respectively, deriving from 3 brains of each genotype. Data are presented as mean ± SEM. *P ≤ 0.05, **P ≤ 0.01 (Mann-Whitney test).

Focusing on the callosal projections, we implanted crystals of DiI in the right somatosensory cortex of sections of P2 brains from WT (N = 7 from 3 brains) and *ArhGAP15*^−/−^ (N = 12 from 4 brains) animals, and examined the length and organization of DiI-labelled projections (callosal) to the contralateral side, after 7 days of diffusion time (Fig. 4A–D). While the general organization was unchanged, in the *ArhGAP15*^−/−^ brains we observed that the median length of DiI+ axons is reduced (−15%, p < 0.001) and the number of fibers reaching the maximum length is reduced (−20%, p < 0.001) (Fig. 4E). In the same sections, we also determined the average length (distance from the midline) of the 5 longest axons, on a minimum of 50 total axons, and found that their length is significantly reduced in the absence of *ArhGAP15* (Fig. 4F). We repeated this analysis at P8 and examined the length of DiI-labelled projection in the contralateral side; also at this age we observed a significant delay.

Next we examined the length and targeting of callosal axons at P45, when the axons have already reached their target. We traced callosal axons length by stereotactically injecting BDA into the right cortex of WT and *ArhGAP15*^−/−^ animals, and examining the contralateral callosal projection 7 days later, by avidin-biotinylated HRP staining. In the two genotypes, BDA+ contralateral callosal projection reached equal distances, as no significant difference was detected (Fig. 4G–K). Collectively, these results indicate that in the absence of *ArhGAP15* pyramidal callosal axons show delayed elongation *in vivo*.

### Retrograde actin flow is increased in the absence of *ArhGAP15*

The small-GTPase Rac1 acts by controlling actin cytoskeleton dynamics at the axonal growth cone, as well as at the leading edge of migrating neuroblasts^27^. Therefore we examined whether the observed inefficient neuritogenesis might be linked to altered actin dynamics at the growth cone of *ArhGAP15*^−/−^ neurons. We established primary cultures of cortical neurons, and electroporated the dissociated cells with RFP-Lifeact^28^, a vector expressing a protein conjugated with RFP and able to bind dynamic actin (a strategy widely used to visualize sites of active actin polymerization/depolymerization in neurons) (Supplementary Fig. S3A,B). Nearly 100% cells stained positive for βIII-tubulin, an early pan-neuronal marker (Supplementary Fig. S3C). Cultures were composed of approximately 92% pyramidal neurons, since staining of both WT and *ArhGAP15*^*−/−*^ cultures with anti-GAD67 detected 8% of GAD67+ neurons (Supplementary Fig. S3D,E).

**Figure 5:**
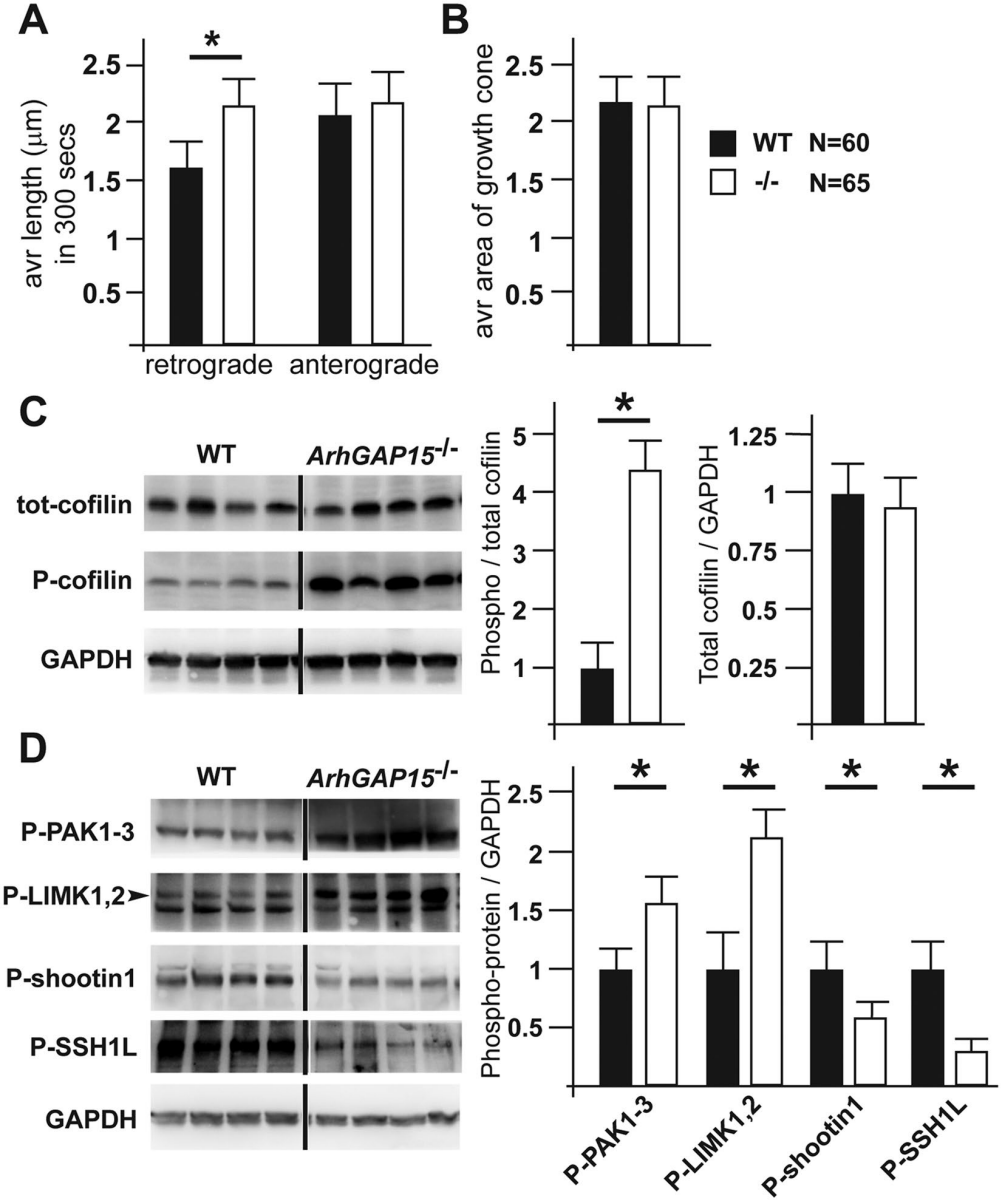
Loss of *ArhGAP15* alters actin dynamics at the axonal growth cone, associated to increased activity of the PAK-LIMK-ADF/Cofilin pathway. (**A**) Quantification of retrograde and anterograde actin filament extension in neurons dissociated from WT or *ArhGAP15*^−/−^ cortices, at E15.5. While anterograde extension is unchanged, in the absence of *ArhGAP15* retrograde actin extension is increased. (**B**) Quantification of the area of the growth cones. The average area is unchanged. N corresponds to the number of neurons analyzed for, respectively, the WT and *ArhGAP15* mutant genotypes, from at least three independent cell cultures. (**C**) Western blot analyses of ADF/cofilin and phospho-ADF/cofilin in total extracts of WT and *ArhGAP15*^−/−^ cortices, at P2. The signal relative to GAPDH is used as loading control and subsequent normalization. On the right, quantification of the ratio of phospho-ADF/cofilin over the amount of total ADF/cofilin and phospho-ADF/cofilin, corrected for GAPDH expression. The WT is placed = 1. (**D**) Western blot analyses of phospho-PAK1-3, phospho-LIMK1/2, phospho-SSHL1 and phospho-shootin1 in total extracts of WT and *ArhGAP15*^−/−^ cortices, at P2. The signal relative to GAPDH is used as loading control and subsequent normalization. On the right, quantification of the ratio of phospho-PAK1-3, phospho-LIMK1/2, phospho-SSHL1 and phospho-shootin1 corrected for GAPDH expression. The WT is considered = 1. Full-length blots are presented in Supplementary Figure S4. Data are presented as mean ± SEM. *P ≤ 0.05 (Student’s t-test).

After 2 DIV, the RFP+ axonal growth cones were subjected to time-lapse video recording, and the raw data were statistically analysed with ImageJ and the dedicated plugins. Considering only the growth cone of the longest neurite (most likely the axon) of each recorded neuron, we observed that in the absence of *ArhGAP15* the retrograde actin movements were significantly increased, while the anterograde flow was not significantly changed (Fig. 5A,B and Supplementary Movies S1 and S2). A significant difference was detected also when we considered separately neurons with a pyramidal-like morphology (based on the cell body) and those (few ones) with a non-pyramidal morphology. Instead, when we examined the growth cones of secondary neurites, no significant alteration was observed. These data strongly indicate that growth cone actin dynamics is altered in *ArhGAP15*^*−/−*^ neurons, suggesting an inverse relationship between actin retrograde flow and axon elongation.

### Loss of *ArhGAP15* results in increased cofilin phosphorylation via the PAK-LIMK pathway

We have previously shown that the genetic disruption of *ArhGAP15* leads to complete absence of the ArhGAP15 protein and that, as consequence of the absence of *ArhGAP15*, Rac1 activity is 1.7-fold (E15.5) and a 2- fold (P2) increased^18,23^. Rho-GTPases are linked to the dynamics of actin cytoskeleton via the control of the actin-binding and actin-severing phosphoprotein ADF/cofilin. We therefore examined the levels of phospho-cofilin in protein extracts from WT or *ArhGAP15*^−/−^ neonatal cortices (P2), by Western blot analyses. In samples from *ArhGAP15*^−/−^ cortices, we observed a significant increase of the phospho-cofilin/total cofilin ratio, while the total cofilin level was unchanged (Fig. 5C). Considering that one of the best studied pathways involved in the phosphorylation (and inactivation) of ADF/cofilin is the Rac-PAK-LIMK^29,30^, we examined the phosphorylation state of LIMK1/2 and PAK1-3. In protein samples from *ArhGAP15*^−/−^ cortices both LIMK1/2 and PAK1-3 were significantly hyperphorphorylated (Fig. 5D). Finally we examined the activation state of slingshot-1L (SSH1L), an ADF/cofilin phosphatase which is activated by phosphorylation. In protein samples from *ArhGAP15*^−/−^ cortices we observed a significant reduction in SSH1L phosphorylation (Fig. 5D), indicating that this phosphatase is hypoactive. In conclusion, Rac1 hyperactivity consequent to the loss of *ArhGAP15*, results in ADF/cofilin inactivation via the increased activity of the PAK-LIMK pathway, and reduced activity of the SSH1L phosphatase.

In order to link the increased ADF/cofilin phosphorylation (hence inactivation) with the increased retrograde actin flow and with shorter and less complex neurites in *ArhGAP15*^−/−^ neurons, we reasoned that loss of *ArhGAP15* may result in decreased phosphorylation of shootin1 and reduced coupling with L1 adhesion. Indeed, increased retrograde flow of the actin network may result from the combined effect of higher actin polymerization and increased myosin II motor function not accompanied by active association of actin-binding proteins with adhesion complexes^9^. Recently, shootin1 phosphorylation downstream of Rac1/Rac3 has been recognized as a key mechanism to couple enhanced actin flow with increased efficacy of adhesion on L1 substrates, needed for actual neurite elongation^31,32^. We examined the phosphorylation state of shootin1 in protein samples from WT and *ArhGAP15*^−/−^ cortices, by Western blot analyses. We observed a significant reduction in the phosphorylation state of shootin1 in *ArhGAP15*^*−/−*^ samples (Fig. 5D). With inactive shootin1, thus an altered engagement of the “clutch” system linking actin filament elongation with L1 dependent adhesion, explains both the increased retrograde actin flow and the inefficient neurite elongation of *ArhGAP15* null neurons.

### Loss of *ArhGAP15* alters synaptic density and causes hyperexitability

**Figure 6:**
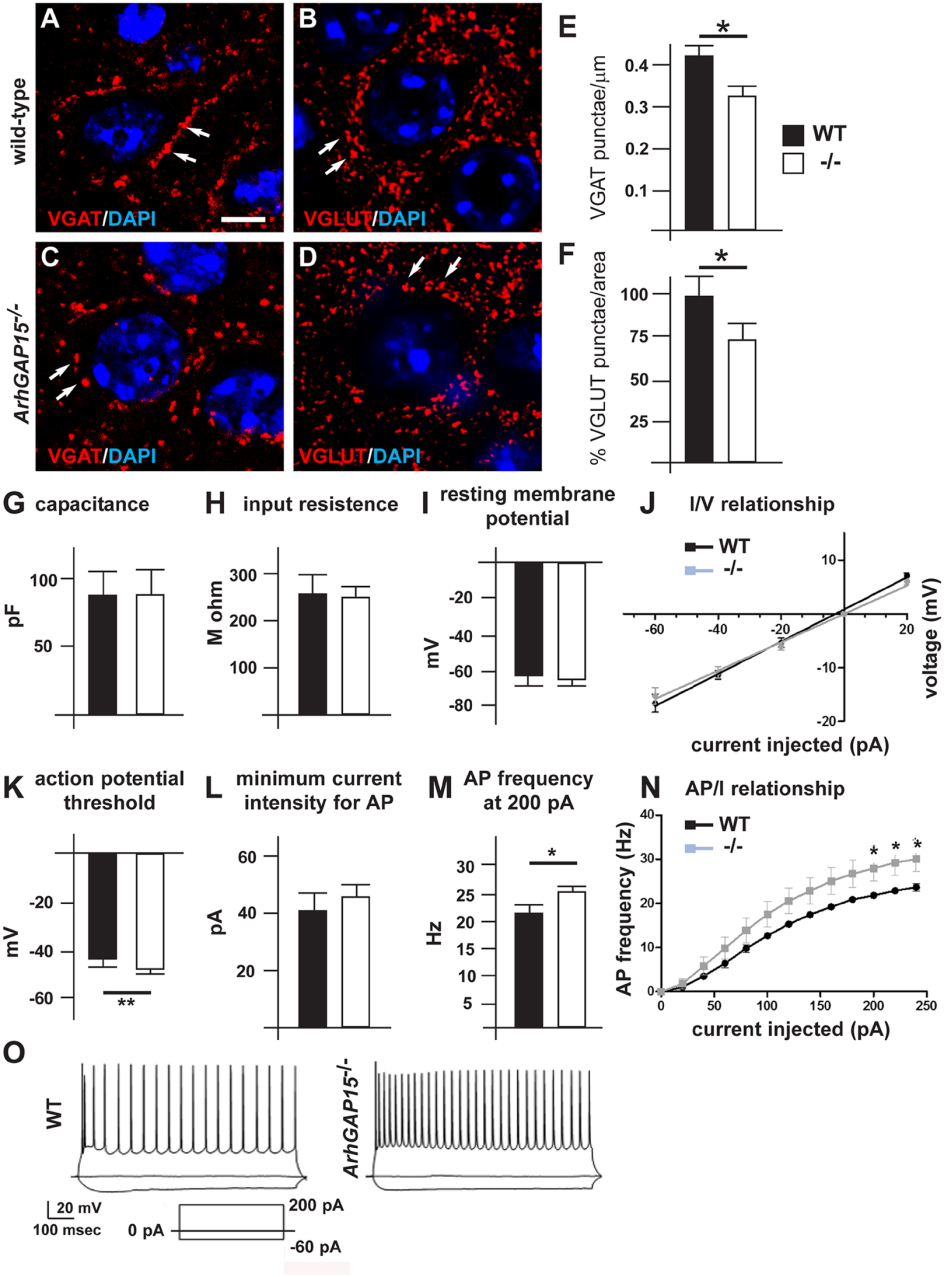
Synapses and electrophysiology of pyramidal neurons in *ArhGAP15*^*−/−*^ mice. (**A**–**D**) Representative images of excitatory and inhibitory synapses on the soma of pyramidal neurons in the somatosensory cortex of WT (**A**,**B**) and *ArhGAP15*^−/−^ (**C**,**D**) brains, at P45. Sections were immunostained for VGAT (left panels, red fluorescence) or for VGLUT (right panels, red fluorescence). Nuclei were counterstained with DAPI. Scale bar in A = 2 μm. (**E**,**F**) Average number of VGAT+ (**E**) and VGLUT (**F**) *punctae* per surface of neuronal soma. WT, solid bars, *ArhGAP15*^*−/−*^, open bars. The density of VGAT+ *punctae* is significantly decreased in the mutant cortex. Asterisks indicate statistical significance (p < 0.01). (**G**–**O**) Electrophysiology of cortical pyramidal neurons. Slices of the somatosensory cortex from WT (open bars) or *ArhGAP15*^*−/−*^ (solid black bars) animals, at P100, were used for recordings. At least 30 neurons were examined per slice. (**G–J**) Passive electrical properties, including the capacitance (**G**), the resistence to input current (**H**), the resting potential (**I**) and the current/voltage ratio (**J**). No differences are observed between the two genotypes. (**K**–**N**) Active electrical properties including the threshold to action potentials (APs) (**K**), the minimal current intensity to induce APs (**L**), the frequency of APs at various currents (200 pA) is shown in (**M**) and the AP/current ratio (**N**). We observe a significantly reduced threshold to APs and an increase frequency of APs at 200 pA. (**O**) Representative traces of WT (left) and *ArhGAP15*^*−/−*^ (right) neurons. *indicates p < 0.05; ***indicates p < 0.001.

Given that Rac GTPases and their regulators have been extensively implicated in early steps of spinogenesis and synaptogenesis^33^, and considering the inefficient neuritogenesis and branching of *ArhGAP15*^−/−^ neurons, we set forth to examine the number of excitatory (VGLUT+) and inhibitory (VGAT+) synapses on the soma of the pyramidal cortical neurons. We determined the density of VGLUT+ and VGAT+ punctae on the perisomatic surface of pyramidal neurons in layer III of the somatosensory cortex of WT and *ArhGAP15*^−/−^ mice at the age P45. We observed a significant reduction (−22.5% in KO vs. WT) of the density of VGLUT+ punctae per equal area (81.9 μm^2^) and a significant reduction of the density of VGAT+ punctae on the pyramidal soma (WT 13/soma ± 0.38 vs. KO 10/soma ± 0.2) (WT 0.41/μm ± 0.023 vs.0.35/μm ± 0.012) (Fig. 6A–F). This result suggests that reduced efficiency of neuritogenesis is associated with an overall reduced efficiency of synaptogenesis onto cortical pyramidal neurons.

To evaluate the functional significance of this, we examined the passive and active electrophysiological properties of cortical pyramidal neurons, comparing brain slices from *ArhGAP15*^−/−^ animals with those from control WT ones. We performed current-clamp recordings from pyramidal neurons in the somatosensory cortex, identified on the basis of their morphological features, of three *ArhGAP15*^−/−^ and three WT males, at the age P100. The passive membrane properties, including capacitance, input resistance and resting potential were unchanged in the *ArhGAP15*^*−/−*^ neurons compared with the WT controls (Fig. 6G–J). On the contrary, in the absence of *ArhGAP15* the action potential threshold was diminished (WT −43.5 ± 1.0 mV, KO −47.7 ± 0.8 mV, p < 0.015) and the action potential frequency at 200 pA of injected current was increased (WT 21.8 ± 0.7 Hz; KO 26.9 ± 2.8 Hz, p < 0.045) in neurons from *ArhGAP15*^−/−^ animals (Fig. 6K–N). These results indicate that cortical pyramidal neurons lacking *ArhGAP15* are more excitable compared to WT neurons.

### Loss of *ArhGAP15* alters the EEG oscillation spectrum

**Figure 7:**
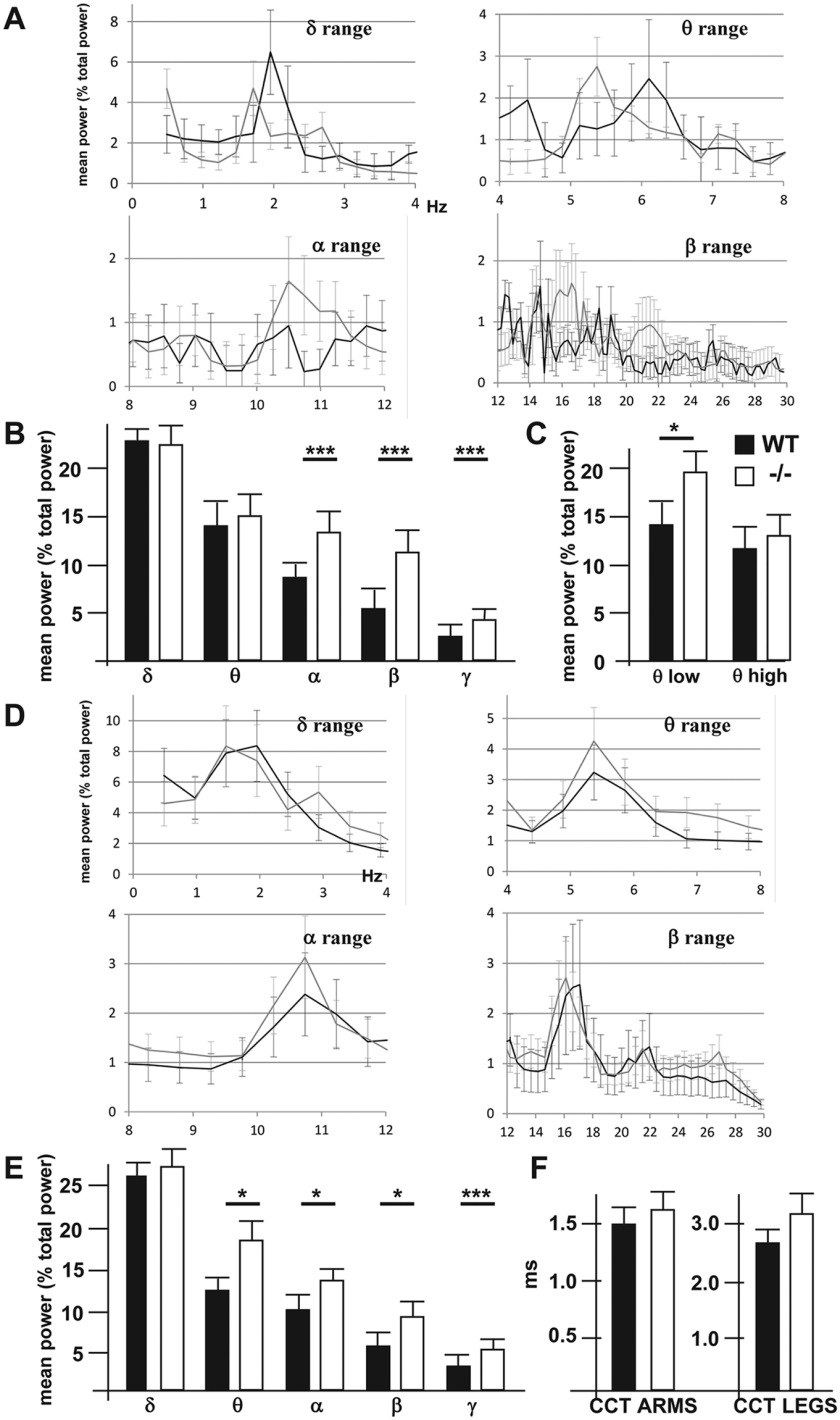
EEG recording from sleeping animals. (**A**) Power spectral profiles of WT (black line) and *ArhGAP15*^−/−^ (grey line) animals, at P40. The power of the individual frequency band (1 Hz bins) was normalized by expressing it as % of total power (1–50 Hz for all epochs). (**B**) Averaged normalized power of each EEG band for WT (solid bars) and *ArhGAP15*^−/−^ (open bars) animals, at the age P40. Significance is reported on the top. (**C**) Quantitative comparison of the θ activity, subdivided in θ-low and θ-high, for WT (solid bars) and *ArhGAP15*^−/−^ (open bars) animals, at the age P40. (**D**) Power spectral profiles of WT (black line) and ArhGAP15^−/−^ (grey line) animals, at P180. The power of the individual frequency band (1 Hz bins) was normalized by expressing it as % of total power (1–50 Hz for all epochs). (**E**) Same as in (**B**), recorded in WT and *ArhGAP15*^−/−^ animals, at P180. (**F**) Central conductance of the brain-to-arm motoneuron path (left) and the brain-to-leg motoneuron path (right) in WT (solid bars) and *ArhGAP15*^−/−^ (open bars) animals, at P45. No difference is detected. Data are presented as mean ± SEM. *P ≤ 0.05, ***P ≤ 0.001 (Student’s t-test).

To evaluate the functional significance of the previous findings, we examined the global electrical activities in the intact brain. In particular, imbalance between excitation and inhibition correlates with changes in power of γ-range oscillations^34,35^ and reduced neuronal complexity seen in mouse models of Huntington Disease is associated with low-shifts of the mean dominant frequency (MDF) in the θ range^36^. In order to confirm our previous findings, we carried out recording of the spontaneous electroencephalogram (EEG) of adult WT and *ArhGAP15*^−/−^ animals at two ages: P40 and P180. At P40, the power spectrum of mutant animals (N = 9), compared to the WT animals (N = 10) showed a significant increase of the relative α- (8–12 Hz), of the β- (12–30 Hz) and of the γ-activity (>30 Hz) (Fig. 7A,B). The overall θ-activity (4–8 Hz) appeared unchanged, however when we subdivided the θ-waves in a high (6–8 Hz) and a low (4–6 Hz) range, we detected a significant increase in the θ low activity in *ArhGAP15*^−/−^ mice (Fig. 7C). No change of the relative power of the δ-activity was observed. In *ArhGAP15*^−/−^ mice we also observed a significant reduction in the variability of the spectral components relative to the β1 activity (Fig. 7A). The MDF changed between groups, with the *ArhGAP15*^−/−^ mice showing a slower MDF than the WT controls in the α range (10.3 vs 11.8 Hz) and in the θ range (5.3 vs 6.1 Hz). At P180 the *ArhGAP15*^−/−^ mice (N = 3 for both genotypes) showed an increase in the relative power of the α, β, and γ frequency oscillations, similar to what we observed in 6-weeks old animals, while no change of the MDF was detected (Fig. 7D,E). To exclude anomalies of the pyramidal motor pathway or other evident neuromuscular defects, in the 6-weeks old WT and *ArhGAP15*^−/−^ mice we examined the peripheral and cortical-to-spinal cord conduction. We did not observe significant changes in the pyramidal conduction relative to the anterior or the posterior limbs (Fig. 7F). All other parameters were found to be in the normal range. In summary, spectral EEG analysis of *ArhGAP15*^*−/−*^ mice shows an increase in the relative power of medium and high frequencies (α, β and γ ranges), a significant increase in the θ low activity (4–6 Hz) and a mild shift of MDF towards lower frequency (α and θ ranges), similar to the mild slowing of background activity seen in most Mowat-Wilson syndrome patients^21,22^. These latter results are in accordance with the reduced complexity of the cortical network previously described.

## Discussion

Hereditary forms of ID in humans are caused by mutations in genes coding for regulators of the Rac1 small-GTPase, or components of the Rac-PAK-LIMK main pathway^37–39^, and recently mutations in Rac1 have been reported in few ID individuals^1^. All these mutations are of loss-of-function type, thus expected to cause hypo-activity of the Rac-PAK-LIMK transduction pathway and aberrant actin dynamics^40^. How altered actin dynamics result in cognitive deficits in human is still largely unclear, although by examining the roles of different genes in many strains of (loss-of-function) mutant mice, it is generally assumed that cognitive deficits are linked to altered synaptic networking and plasticity, as well as excitation/inhibition imbalance^7,40,41^.

One aspect that has remained poorly studied is whether any misregulation of Rac activity, e.g. hyper- or hypo-, would alter actin dynamics in ways that present common features and thus result in related cognitive deficits. There are substantial similarities with the phenotypes of mice with a conditional loss of *Rac1* or of *Rac1*+ *Rac3*; specifically the single and combined *Rac1/Rac3* mutant animals show defective axonal elongation, altered synaptic density and plasticity, associated with memory and learning deficits^10,13,40^, similar to the *ArhGAP15* ones. This observation indicates that the hypo-activity and the hyper-activity of Rac1 can induce similar neuronal defects, and suggests that a fine regulation of the Rac1 activity is necessary to attain the full complexity and functioning of cortical neuronal network.

Considering that, in human, mutations in *Oligophrenin1* (a Rho GAP) cause X-linked ID^42,43^, and that the loss of *ArhGAP15* (a Rac GAP) is linked to rare variants of the Mowat-Wilson disease with severe neurologic and cognitive deficits^19^, here we examined a mouse model in which Rac1 is hyperactive as the consequence of loss of the Rac-specific GAP protein *ArhGAP15*. We observe delayed axonal lengthening, reduced neuronal complexity and altered synaptic density on cortical pyramidal neurons, associated with their mild hyperexcitability. We also observe an increase in the relative power in the α, β, and γ ranges, accompanied by cognitive/learning deficits in the adult animals^18^. Frequency oscillations could reflect disturbed neuronal synchronization, e.g., caused by a dysfunctional GABA/glutamate system^44^. Altogether, the synchronous occurrence of θ, α, β and γ activity indicates the existence of distributed oscillatory systems which are linked with sensory and cognitive functions^45^. Of interest, a diminished cortical γ activity has been linked to impaired inhibitory input from fast-spiking parvalbumin-expressing neurons^46^, and we observe indeed a reduced density of GABA synapses. On the contrary, an increased γ activity has been linked to increased excitability of pyramidal neurons which enhances the inhibitory function of parvalbumin neurons via local circuits^34^. In the absence of *ArhGAP15* this second mechanism seems prevalent, although the number, distribution and activity of inhibitory neurons in the *ArhGAP15*^−/−^ cortex will have to be examined in details.

Recent studies have demonstrated that GAP proteins, by modulating the Rac1 activity, regulate nearly all aspects of brain development, being essential for the regulation of neurogenesis, neuronal morphology, axon growth, synapse formation and plasticity^47,48^. Indeed mutations in GAP molecules (specific for Rho-class GTPases) cause axonal, dendritic and synaptic defects. Rho-class GTPases, control actin cytoskeleton reorganization in complex ways^49^. The best studied pathway involves the PAK1/3-dependent phosphorylation of LIMK and consequent phosphorylation (and inactivation) of ADF/cofilin^29,30^. Protein kinases and phosphatases responsible for cofilin phosphorylation and dephosphorylation play crucial roles in regulating actin cytoskeleton reorganization^50^, and indeed the levels of cofilin phosphorylation drastically change in response to various extracellular stimuli that affect actin dynamics, cell migration and morphogenesis. Here we show that Rac hyperactivity results in a higher level of ADF/cofilin phoshorylation via the increased activity of the PAK-LIMK pathway, and reduced activity of the SSH1L phosphatase. Increased ADF/cofilin phosphorylation, usually associated with reduced actin dynamics^50^, can explain the inefficient neuritogenesis and branching that we observed in the absence of *ArhGAP15*.

We show that in the absence of *ArhGAP15* retrograde actin flow at the growth cone is increased. This should facilitate neurite formation^51^, however this occurs upon coupling of actin retrograde flow with cell adhesion molecules (CAMs) via an efficient linking bridge, known as “clutch”^31,52^. Among the molecules participating in the “clutch”, shootin1 is a brain-specific protein that interacts both with the F-actin network and with L1-CAM in axonal growth cones^31,53^. Shootin1 phosphorylation enhances its interaction with the actin network, promoting filopodia and axon extension^32,54,55^. On the contrary, silencing or hypophosphorylation of shootin1 uncouples the actin flow from membrane adhesion and inhibits neurite elongation^32,52,56^. In the absence of *ArhGAP15*, we observe reduced shootin1 phosphorylation indicating that, in the presence of hyperactive Rac1, shootin1 fails to efficiently couple the F-actin flow with cell adhesion, and this “clutch disengagements” results in shorter and simpler neurites.

Current knowledge on the role of Rac1 for correct neuronal positioning, wiring and connectivity derives largely from the use of either constitutively active (CA) or dominant-negative (DN) Rac1 variants, or the generation of transgenic animals^9,11,15,57^. Such an approach, however, does not provide the ideal experimental setting to study the function of the GTPase. In fact, CA forms require continuous GTP-binding/GTP-hydrolysis cycling for effective signaling flow of the GTPase, while DN forms drastically interfere with endogenous functions. Likewise, the elimination of GTPases, such as their genetic knockouts, alters their amount but not their activity. Not surprisingly *in vitro* and *in vivo* results have led to contradictory conclusions. For example, hyperactive cdc42 *in vitro* promotes dendritic arbor growth^58^, while the deletion of the cdc42-GAP *NOMA-GAP in vivo* results in hyperactive cdc42 and reduced (not increased) complexity of dendritic arborization^59^. Thus, the approaches of directly interfering or eliminating Rac1 appear inadequate to fully comprehend the physiological role of active vs. inactive GTPases and the neuronal bases of human ID in the presence of mutant GTPase regulators. Instead, manipulating Rac1 regulators offers a better opportunity to alter Rac1 activity in ways that more closely recapitulates the condition of hereditary forms of ID.

Defining the impact of modest misregulation of GTPases activity on the construction, function and plasticity of cortical networks is a main scientific and medical issue, that will require addressing the role of each neuronal-specific GTPase regulator in improved models, such as human iPSC-derived neurons and brain organoids. Relevant information may open ways to pharmacologically modulate Rac activity for therapeutic purposes^40^.

## Materials and Methods

### Mouse strains

All animal procedures were approved by the Animal Ethics Committee of the University of Torino and by the Ministry of Health. Animals were maintained according to institutional animal welfare guidelines and legislation, under veterinarian surveillance. The *ArhGAP15*^−/−^ mouse strain has been previously described^23^. The targeted mutation disrupts the first coding exon by inserting a *lacZ* reporter cassette and replacing it with the neomycin-resistance cassette. *ArhGAP15*^+/−^ mice were backcrossed in C57BL/6 J for five generations before generating homozygous. Heterozygous and homozygous mutant mice are born at normal mendelian frequency, appear overall normal, are viable and fertile, could feed and mate at regular rates, and did not show evident neurological or motor impairments^23^. Animals were maintained in a mixed C57/BL6:DBA genetic background.

### Brain preparation for histological analysis

For embryo collection, the day of the vaginal plug was considered as embryonic age 0.5. Embryos were collected by caesarean section from anesthetized pregnant dams, transferred in PBS, and either dissociated for neuronal cultures, or fixed in cold 4% PFA for 8–12 hrs and processed for cryopreservation and histology. Extra-embryonic tissues were used for genotyping by PCR.

For the collection of postnatal and adult brains, WT and *ArhGAP15*^−/−^ mice were anesthetized with Avertin (for each animal, 30 μl pure Avertin in 400 μl PBS), transcardially perfused with 5 mM sodium phosphate-buffered 0.9% saline (PBS, pH 7.4) and then with 2% formaldehyde, pH 7.0 (adjusted with NaOH). After the mice were left thus for 2 hrs, their brains were removed, immersed in PBS, and kept in a solution of 30% sucrose in PBS for cryoprotection. The blocks were cut into 25 μM thick coronal sections on a freezing microtome at −20 °C; they were collected in multi-well plates and immersed in PBS.

WT and *ArhGAP15*^−/−^ females were mated respectively with WT and ArhGAP15^−/−^ males; pregnant dams were anesthetized as described above and embryos were collected by caesarean section at the embryonic day 15.5 (E15.5). Embryonic heads were post-fixed overnight; then heads were washed in PBS and kept in a solution of 30% sucrose in PBS for cryoprotection, cut into 20 μm-thick coronal sections and collected on super-adhesive glass slides.

For the histoenzymatic detection of βgal expression, brains specimens were fixed briefly with cold 4% PFA, washed with PBS several times, then included in 4% agarose, vibratome-sectioned (150 μm-thick). Sections were collected on slides and stained with the chromogenic substrate Xgal, according to standard methods.

### Antibodies and immunostaining

Sections of embryonic brains (25 μm-thick) or adult brains (40 μm-thick) were collected and stored at −20 °C. For immunofluorescence, sections are incubated for 1 hr at R.T. in a solution composed by 4% Bovine Serum Albumin (BSA) in PBS, 10% Goat Serum and PBS 0.5% TritonX-100.

Primary antibodies were incubated in a solution composed by 0.8% BSA in PBS, 2% Goat Serum and PBS 0.1% TritonX-100, overnight at 4 °C, followed by three rinses with PBS for 10 min.

Sections were incubated for 2 hrs with secondary antibodies in PBS, followed by three rinses with PBS for 10 min. Finally sections were mounted onto coated super-adhesive glass slides and covered with MOWIOL for microscopic observation. For photodocumentation, we used a Leica SP5 confocal microscope with Z-sections of 0.5 μm. Raw images were digitally processed to normalize the background and optimize the contrast, rotated and sized with Photoshop (Adobe).

The following primary antibodies were used: rabbit anti-Cux1 (Santa Cruz, used 1/100), rat anti-Ctip2 (Abcam, used 1/500), mouse anti-Satb2 (Abcam, used 1/100), rabbit anti-Tbr1 (Abcam, used 1/400), chicken anti-β-galactosidase (βGal, Aves, used 1/1000), rabbit anti-Vescicular GABA Transporter (VGAT, Synaptic System, used 1/1000), guinea pig anti-Vescicular GLUT Transporter (VGLUT, used 1/5000). Secondary antibodies were Alexa Fluor 488 donkey anti-chicken IgG, AlexaFluor 568 goat anti-rat IgG, AlexaFluor 568 goat anti-rabbit IgG, AlexaFluor 647 goat anti-rabbit IgG (Invitrogen, used at 1/400).

### *In situ* hybridization

Perfused brains were post-fixed overnight, rinsed in RNAse-free PBS, cryoprotected in 30% sucrose for 24 hrs, embedded/frozen in OCT and sectioned at 20 μM thickness. Hybridization was performed with DIG-labeled riboprobes corresponding to the antisense sequence of the murine *ArhGAP15* cDNA or of bacterial *lacZ*. Sections were permeabilized with 3 μg/ml proteinase K, washed in PBS, and acetylated with 1.3% triethanolamine and 0.25% acetic anhydrate at R.T. Sections were prehybridized in 50% formamide at 60 °C, hybridized with the DIG-labeled probes for 16 hrs, washed, incubated with an anti-DIG-AP antibody (Roche), and developed with NBT-BCIP (Sigma).

### Western blot analyses

Twenty-four hours after transfection, cells were lysed in 100 μl of loading buffer (2% sodium dodecyl sulfate, 30% glycerol, 300 mM β-mercaptoethanol, 100 mM Tris-HCl pH 6.8): extracts were separated on SDS-10% polyacrylamide gels, transferred and incubated with the relative antibodies and developed according to the manufacturer’s instructions (GeneSpin). The following antibodies were used: anti-ADF/cofilin mouse monoclonal antibody (Abcam); anti-ADF/cofilin phosphoserine 3, rabbit polyclonal antibody (Cell Signalling Technology); anti-phospho-LIMK 1/2 (Tyr507/Thr508), rabbit polyclonal antibody (Millipore); anti-phospho-PAK1/2/3 (Ser141), rabbit polyclonal antibody (Invitrogen). The antibody detecting total and phospho-slingshot was kindly provided by Dr. Nicklaus Sparrow (Univ. Central Florida, Orlando USA)^60^. Secondary antibodies were conjugated anti-mouse and anti-rabbit (Santa Cruz).

Rac1/Rac3 activity was determined by pull-down assays using a glutathione-S-transferase-PAK-CD (PAK-CRIB domain) fusion protein, containing the Rac binding region from human PAK1. Lysates of the embryonic cortices were centrifuged at 4 °C for 10 min at 13,000 RPM, and the supernatant was incubated with glutathione S-transferase PAK glutathione-coupled Sepharose 4B beads (GE Healthcare) for 30 min. at 4 °C. Proteins bound to the beads were washed 3 times in lysis buffer, and then quantified by polyacrylamide gel electrophoresis and Western blot analysis using an anti-Rac1 antibody (Upstate Biotechnology; Lake Placid, USA). An anti-ArhGAP15 monoclonal antibody was also used, this was raised in mouse against a GST-fusion protein of an ArhGAP15 peptide spanning from aminoacids 220–320. Images were quantified by densitometric analysis using Quantity One software (BioRad, CA, USA). The G-LISA colorimetric assay (Cytoskeleton Inc, CO USA) was used according to the manufacturer’s instructions.

### Primary cultures of cortical neurons

WT, *ArhGAP15*^+/−^ or *ArhGAP15*^−/−^ embryos at the age E15.5 were used to establish primary cultures of cortical neurons. Embryonic heads were dissected in sterile conditions in cold Leibovitz’s L-15 Medium (Gibco, Life Technologies), the cortices were dissected free of the rest of the brain, deprived of the meninges and dissociated. For the dissociation, Neurobasal B27 (Neurobasalmedium, Glutamine 1/100, B27 1/50, Gentamicine 1/1000) was used, first with mechanical shearing and then by adding trypsin for 15 minutes at 37 °C, followed by centrifugation and resuspension in culture medium. Two cellular pools, WT and *ArhGAP15*^−/−^, were thus obtained. Each pool of cell suspension was quantified through Countess (Life Technologies) and 7 × 10^6^ cells from each pool was nucleofected (i.e. electroporated) with PGK-eGFP vector (2 μg) or with the LifeAct-RFP vector (2 μg)^28^ using Nucleofector 2b (Nucleofector System, Lonza, Basel, Switzerland) with the electroporation code O-005^61^.

Nucleofected neurons were plated on round glasses placed in 3.5 cm diameter Petri dishes, previously coated with poly-L-lysine (1 mg/ml; Sigma; 1/10 dilution) at the density of 10^6^/cm^2^, and allowed to adhere. After cells attach to the glasses, medium was replaced with fresh plating medium to remove unattached cells and neurons were incubated for 3 days at 37 °C in 5% CO_2_ saturation atmosphere. Cells were observed during the three days in order to verify their culture condition and the absence of contaminations; then culture medium was aspirated and cells were washed with sterile PBS; cells were fixed for 20 min. with PFA 4% and washed again in PBS. Round glasses with adherent cortical primary neurons were picked up with tweezers, laid on microscopy glass, then mounted with MOWIOL and photographed.

Fluorescence images were acquired using an inverted microscope (Axio Observed Z1, Zeiss), ApoTome system. Images were digitally captured using a cooled 16-bit camera (Axio MRM, Zeiss) with Axio Vision Release 4.7.1 software.

### Morphological analyses

Cultured WT and *ArhGAP15*^*−/−*^ neurons were classified for their morphological features as unipolar, bipolar and multipolar, based on the presence of 1, 2 or >2 main neurites stemming from the cell body, as shown in Supplementary Figure S2. Analyses were performed with the ImageJ informatics suite, and the ImageJ plugin Cell Counter. Cultured WT and *ArhGAP15*^*−/−*^ neurons were then analyzed for the main neurite length, the number of secondary neuritis, and the number and distribution of branches (indicating the arborization rate): together these criteria define the complexity of neuronal structure. A minimum of 150 WT and *ArhGAP15*^−/−^ neurons were counted. To perform this analysis, we used the basic functions of ImageJ software^62^, measuring the length of the main neurite of each neuron and the number of secondary neurites, departing from the main neurite. Arborization of each neuron was quantified by Sholl analysis^63^, a computer-assisted method uses concentric circles around the neuronal soma, and determines how many times each neuron intersects each of these circles (plugin ImageJ).

The same methods were applied to examine fluorescent images of individual neurons stained with the DiI retrograde tracing method (see below). For these analyses, at least 50 neurons belonging to the same cortical area and layer were traced for each genotype with the Neurolucida software package. Neurons were analyzed using the Neurolucida Explorer software package (MBF Bioscience) quantifying the length of axon, dendrites and apical dendrite, and making the Sholl analysis.

### Anterograde tracing of cortical callosal axons

We used a procedure described^64^. WT and *ArhGAP15*^−/−^ animals at the age P2 were transcardially perfused with 2% PFA, removed from the skull and post-fixed for at least 7 days. Crystals of 1, 1′-Dioctadecyl-3, 3, 3′, 3′-Tetramethylindocarbocyanine Perchlorate (DiI, size 25 μm) (from Molecular Probes) were inserted with a fine needle in the right somatosensory cortex (AP = +0.8; ML = +1.5; DV = −0.5), and the dye was allowed to diffuse for 7 days at 37 °C. Mice at the age P8 were subjected to the same procedure, with the exception of the diffusion time which was extended to 14 days. Thick (100 μm) vibratome sections were then examined with a fluorescent microscopy. Images were used to computer assisted analyses to estimate the maximum and the average length of the callosal axons. Seven and 12 sections (from at least 3 independent brains) were examined for, respectively, the WT and the *ArhGAP15* mutant genotypes. The fluorescence intensity (F.I.) and the thickness (T) of the callosal bundle of axons was measured at various distances (from 0 to 1612 μm) from the callosal commissure, set as distance = 0. The multiplication F.I.*T was calculated at each distance (F.I.*T-d), and the results were expressed as F.I.*T-d relative to the F.I.*T at distance = 0. The length of the five longest axons was also determined, on a minimum of 50 axons from at least 3 independent brain samples for each genotype. At the age P45, cortical neurons were labeled by pressure injecting 0.5 μL of Biotinylated Dextran Amine (BDA, Thermo Fisher) MW 10 000 (5% in 0.01 M phosphate buffer)^65^ into the right cortex of anesthetized WT and *ArhGAP15*^−/−^ animals, the mice were let recover and maintained in standard conditions for 7 days. Animals were then transcardially perfused, the brains were collected and cryosectioned (40 μM thick). Floating sections were stained with avidin-biotinylated HRP procedure and standard DAB reaction, and mounted on glass slides.

### Time-Lapse video recordings

Dissociated cortical neurons from E15.5 WT and *ArhGAP15*^−/−^ embryos were prepared and nucleofected with the Life-act vector. After 2 DIV individual growth cones were examined by fluorescent time-lapse video. Time lapses were recorded for 5 minutes with an interval of 10 seconds using a 40X PlanApo N.A. 1.4 oil immersion objective on the cells kept in the microscope incubator at 37 °C and 5% CO_2_. The acquired movies (N = 60 and 65 good quality ones, for WT and *ArhGAP15*^−/−^, respectively) were used to determine the total anterograde and retrograde actin movements, using ImageJ dedicated plugins. Briefly, for each growth cone a kimograph was first extracted, then the movements parameters were computed and exported for statistical analyses.

### Electrophysiology

WT and *ArhGAP15*^−/−^ mice were anesthetized in a chamber saturated with chloroform-saturated chamber and decapitated, the brain was rapidly removed and placed in an ice-cold solution containing (in mM) 220 sucrose, 2 KCl, 1.3 NaH_2_PO_4_, 12 MgSO_4_, 0.2 CaCl_2_, 10 glucose, 2.6 NaHCO_3_ (pH 7.3, equilibrated with 95% O_2_ and 5% CO_2_). Coronal brain slices (300 μm-thick) were prepared with a vibratome VT1000 S (Leica), incubated first for 40 min at 37 °C and then for 30 min at R.T. in artificial CSF (ACSF) consisting of (in mM) 126 NaCl, 3 KCl, 1.25 NaH_2_PO_4_, 1 MgSO_4_, 2 CaCl_2_, 25 glucose, and 26 NaHCO_3_ (pH 7.3, equilibrated with 95% O_2_ and 5% CO_2_). Slices were transferred to a recording chamber perfused with ACSF at a rate of ∼2 ml/min and at R.T. Whole-cell patch-clamp electrophysiological recordings were performed with a Multiclamp 700 B amplifier (Axon CNS molecular devices, USA) and using an infrared-differential interference contrast microscope. Patch microelectrodes (borosilicate capillaries with a filament and an outer diameter of 1.5 μm; Sutter Instruments) were prepared with a four-step horizontal puller (Sutter Instruments) and had a resistance of 3–5 MΩ.

Current-clamp analysis were performed on pyramidal neurons of the somatosensory cortex, identified by their morphological features, under video microscopy with an internal solution containing (in mM) 126 K-gluconate, 4 NaCl, 1 EGTA, 1 MgSO_4_, 0.5 CaCl_2_, 3 ATP (magnesium salt), 0.1 GTP (sodium salt), 10 glucose, 10 HEPES-KOH (pH 7.28; osmolarity adjusted to 280 mOsm). A series of current steps (from −60 pA to 240 pA) were injected to induce action potentials (20-pA injection current per step, duration of 1 s) in order to study passive and active properties of the neuron membrane. Data analysis was performed offline with Clampfit 10.1 software.

### EEG recordings

Three-months old WT and *ArhGAP15*^−/−^ females (in C57BL6) were maintained in the same environmental conditions, then anaesthetized with Avertin and used for EEG recording. Two Ag-AgCl ball electrode were placed over the left neocortex, the common reference electrode was fixed on the right pinna. The recording of the ECG activity was performed using a 16-channel data acquisition system (PowerLab 16/30, AD Instruments, Australia). The biological signals from the electrodes were amplified and filtered (0.1–50 Hz bandpass) using BioAmp amplifiers (AD Instruments). The EcoG signal was digitalized at a sampling rate of 1024, then displayed and stored. Only the spikes with amplitude greater than three-fold the baseline were included. The EEG power spectrum was analysed with LabChart 7.2 software (AD Instruments). FFT analysis was applied for at least 5 min time windows of continuous noise-free segments. FFTs were collected as power spectra with a sample size of 2048 (without overlap). EEG power was computed in the selected frequency ranges: δ range (0.5–4 Hz), θ range (4–8 Hz), α range (8–12 Hz), β range (12–30 Hz) and γ range (30–50 Hz). To normalize the data, EEG power (μV^2^) for each frequency bin within each epoch was calculated as a percentage of the total EEG power (1–56 Hz) of all epochs.

## Electronic supplementary material

Supplementary Information

video WT neuron

video ArhGAP15 KO neuron

## References

[1] Lelieveld, S. H. *et al*. Meta-analysis of 2,104 trios provides support for 10 new genes for intellectual disability. *Nat. Neurosci*. 10.1038/nn.4352 (2016). 10.1038/nn.4352 27479843

[2] Reijnders MRF (2017). RAC1 Missense Mutations in Developmental Disorders with Diverse Phenotypes. Am. J. Hum. Genet..

[3] Maglorius Renkilaraj MRL (2017). The intellectual disability protein PAK3 regulates oligodendrocyte precursor cell differentiation. Neurobiol. Dis..

[4] Klein KM (2017). The phenotypic spectrum of ARHGEF9 includes intellectual disability, focal epilepsy and febrile seizures. J. Neurol..

[5] Ramakers GJA (2012). Dysregulation of Rho GTPases in the αPix/Arhgef6 mouse model of X-linked intellectual disability is paralleled by impaired structural and synaptic plasticity and cognitive deficits. Hum. Mol. Genet..

[6] Watabe-Uchida M, John KA, Janas JA, Newey SE, Van Aelst L (2006). The Rac activator DOCK7 regulates neuronal polarity through local phosphorylation of stathmin/Op18. Neuron.

[7] Murakoshi H, Wang H, Yasuda R (2011). Local, persistent activation of Rho GTPases during plasticity of single dendritic spines. Nature.

[8] Vitriol EA, Zheng JQ (2012). Growth Cone Travel in Space and Time: The Cellular Ensemble of Cytoskeleton, Adhesion, and Membrane. Neuron.

[9] Gomez TM, Letourneau PC (2014). Actin dynamics in growth cone motility and navigation. J. Neurochem..

[10] Vaghi V (2014). Rac1 and Rac3 GTPases control synergistically the development of cortical and hippocampal GABAergic interneurons. Cereb. Cortex.

[11] Tivodar S (2015). Rac-GTPases Regulate Microtubule Stability and Axon Growth of Cortical GABAergic Interneurons. Cereb. Cortex.

[12] Corbetta, S. *et al*. Hyperactivity and novelty-induced hyperreactivity in mice lacking Rac3. *Behav Brain Res* **186**, 246–55, OD–2007/09/25 (2008). 10.1016/j.bbr.2007.08.019 17889944

[13] Corbetta, S. *et al*. Essential role of Rac1 and Rac3 GTPases in neuronal development. *FASEB J* **23**, 1347–57, OD–2009/01/08 (2009). 10.1096/fj.08-121574 PMC7617009 19126596

[14] Pennucci, R., Tavano, S., Tonoli, D., Gualdoni, S. & de Curtis, I. Rac1 and Rac3 GTPases regulate the development of hilar mossy cells by affecting the migration of their precursors to the hilus. *PLoS One* **6**, e24819, OD-2011/09/29 (2011). 10.1371/journal.pone.0024819 PMC3176786 21949760

[15] de Curtis, I. Roles of Rac1 and Rac3 GTPases during the development of cortical and hippocampal GABAergic interneurons. *Front Cell Neurosci* **8**, 307, OD-2014/10/14 (2014). 10.3389/fncel.2014.00307 PMC4174739 25309333

[16] Govek EE (2004). The X-linked mental retardation protein oligophrenin-1 is required for dendritic spine morphogenesis. Nat Neurosci.

[17] Seoh ML, Ng CH, Yong J, Lim L, Leung T (2003). ArhGAP15, a novel human RacGAP protein with GTPase binding property. FEBS Lett..

[18] Zamboni, V. *et al*. Disruption of ArhGAP15 results in hyperactive Rac1, affects the architecture and function of hippocampal inhibitory neurons and causes cognitive deficits. 1–17, 10.1038/srep34877 (2016). 10.1038/srep34877 PMC5054378 27713499

[19] Smigiel R (2010). Severe clinical course of Hirschsprung disease in a Mowat-Wilson syndrome patient. J Appl Genet.

[20] Mulatinho MV (2012). Severe intellectual disability, omphalocele, hypospadia and high blood pressure associated to a deletion at 2q22.1q22.3: case report. Mol Cytogenet.

[21] da Paz JA, Kim CA, Goossens M, Giurgea I, Marques-Dias MJ (2015). Síndrome de Mowat-Wilson: Estudo neurológico e molecular em sete pacientes. Arq. Neuropsiquiatr..

[22] Cordelli DM (2013). Epilepsy in Mowat-Wilson syndrome: Delineation of the electroclinical phenotype. Am. J. Med. Genet. Part A.

[23] Costa C (2011). The RacGAP ArhGAP15 is a master negative regulator of neutrophil functions. Blood.

[24] Radu, M. *et al*. ArhGAP15, a Rac-specific GTPase-activating protein, plays a dual role in inhibiting small GTPase signaling. *J Biol Chem* **288**, 21117–25, OD–2013/06/14 (2013). 10.1074/jbc.M113.459719 PMC3774378 23760270

[25] Huang Y (2013). Expression of transcription factor Satb2 in adult mouse brain. Anat. Rec. (Hoboken)..

[26] Bedogni F (2010). Tbr1 regulates regional and laminar identity of postmitotic neurons in developing neocortex. Proc. Natl. Acad. Sci. USA.

[27] Sayyad WA (2016). The Role of Rac1 in the Growth Cone Dynamics and Force Generation of DRG Neurons. PLoS One.

[28] Riedl J (2008). Lifeact: a versatile marker to visualize F-actin. Nat Methods.

[29] Jacobs T (2007). Localized activation of p21-activated kinase controls neuronal polarity and morphology. J. Neurosci..

[30] Delorme-walker VD (2011). Pak1 regulates focal adhesion strength, myosin IIA distribution, and actin dynamics to optimize cell migration. J Cell Biol..

[31] Shimada T (2008). Shootin1 interacts with actin retrograde flow and L1-CAM to promote axon outgrowth. J Cell Biol.

[32] Toriyama M, Kozawa S, Sakumura Y, Inagaki N (2013). Conversion of a signal into forces for axon outgrowth through Pak1-mediated shootin1 phosphorylation. Curr Biol.

[33] Martin-Vilchez S (2017). RhoGTPase Regulators Orchestrate Distinct Stages of Synaptic Development. PLoS One.

[34] Stroganova TA (2015). Altered modulation of gamma oscillation frequency by speed of visual motion in children with autism spectrum disorders. J. Neurodev. Disord..

[35] Siegel M, Donner TH, Engel AK (2012). Spectral fingerprints of large-scale neuronal interactions. Nat. Rev. Neurosci..

[36] Fisher SP (2016). Quantitative Electroencephalographic Analysis Provides an Early-Stage Indicator of Disease Onset and Progression in the zQ175 Knock-In Mouse Model of Huntington’s Disease. Sleep.

[37] De Filippis B, Romano E, Laviola G (2014). Aberrant Rho GTPases signaling and cognitive dysfunction: *in vivo* evidence for a compelling molecular relationship. Neurosci. Biobehav. Rev..

[38] Ba W, van der Raadt J, Nadif Kasri N (2013). Rho GTPase signaling at the synapse: Implications for intellectual disability. Exp. Cell Res..

[39] Ba W (2016). *TRIO* loss of function is associated with mild intellectual disability and affects dendritic branching and synapse function. Hum. Mol. Genet..

[40] Tejada-Simon MV (2015). Modulation of actin dynamics by Rac1 to target cognitive function. J. Neurochem..

[41] De Filippis B (2015). Modulation of Rho GTPases rescues brain mitochondrial dysfunction, cognitive deficits and aberrant synaptic plasticity in female mice modeling Rett syndrome. Eur. Neuropsychopharmacol..

[42] Barresi S (2014). Oligophrenin-1 (OPHN1), a Gene Involved in X-Linked Intellectual Disability, Undergoes RNA Editing and Alternative Splicing during Human Brain Development. PLoS One.

[43] Khelfaoui M (2009). Inhibition of RhoA pathway rescues the endocytosis defects in Oligophrenin1 mouse model of mental retardation. Hum Mol Genet.

[44] Karch S (2016). Increased Event-Related Potentials and Alpha-, Beta-, and Gamma-Activity Associated with Intentional Actions. Front. Psychol..

[45] Başar E, Başar-Eroğlu C, Karakaş S, Schürmann M (2000). Brain oscillations in perception and memory. Int. J. Psychophysiol..

[46] Bartos M, Vida I, Jonas P (2007). Synaptic mechanisms of synchronized gamma oscillations in inhibitory interneuron networks. Nat. Rev. Neurosci..

[47] Yang C, Kazanietz MG (2007). Chimaerins: GAPs that bridge diacylglycerol signalling and the small G-protein Rac. Biochem. J..

[48] Bacon C, Endris V, Rappold GA (2013). The cellular function of srGAP3 and its role in neuronal morphogenesis. Mech. Dev..

[49] Hua ZL, Emiliani FE, Nathans J (2015). Rac1 plays an essential role in axon growth and guidance and in neuronal survival in the central and peripheral nervous systems. Neural Dev..

[50] Mizuno K (2013). Signaling mechanisms and functional roles of cofilin phosphorylation and dephosphorylation. Cell Signal.

[51] Flynn KC (2012). ADF/Cofilin-Mediated Actin Retrograde Flow Directs Neurite Formation in the Developing Brain. Neuron.

[52] Cooper JA (2013). Mechanisms of cell migration in the nervous system. J Cell Biol..

[53] Santiago-Medina, M., Gregus, K. A. & Gomez, T. M. PAK–PIX interactions regulate adhesion dynamics and membrane protrusion to control neurite outgrowth. *J. Cell Sci*. **126** (2013). 10.1242/jcs.112607 PMC3635460 23321640

[54] Le Clainche C, Carlier M-F (2008). Regulation of Actin Assembly Associated With Protrusion and Adhesion in Cell Migration. Physiol. Rev..

[55] Lowery LA, Vactor DV (2009). The trip of the tip: understanding the growth cone machinery. Nat. Rev. Mol. Cell Biol..

[56] Marsick BM, San Miguel-Ruiz JE, Letourneau PC (2012). Activation of ezrin/radixin/moesin mediates attractive growth cone guidance through regulation of growth cone actin and adhesion receptors. J. Neurosci..

[57] Koh CG (2006). Rho GTPases and their regulators in neuronal functions and development. Neurosignals.

[58] Newey SE, Velamoor V, Govek E-E, Van Aelst L (2005). Rho GTPases, dendritic structure, and mental retardation. J. Neurobiol..

[59] Rosário M (2012). Neocortical dendritic complexity is controlled during development by NOMA-GAP-dependent inhibition of Cdc42 and activation of cofilin. Genes Dev..

[60] Sparrow N (2012). The Actin-Severing Protein Cofilin Is Downstream of Neuregulin Signaling and Is Essential For Schwann Cell Myelination. J. Neurosci..

[61] Zeitelhofer, M., Vessey, J. P., Thomas, S., Kiebler, M. & Dahm, R. Transfection of cultured primary neurons via nucleofection. *Curr Protoc Neurosci* Chapter 4, Unit 432 (2009). 10.1002/0471142301.ns0432s47 19340811

[62] Schneider CA, Rasband WS, Eliceiri KW (2012). NIH Image to ImageJ: 25 years of image analysis. Nat Methods.

[63] SHOLL DA (1953). Dendritic organization in the neurons of the visual and motor cortices of the cat. J. Anat..

[64] Vercelli A, Repici M, Garbossa D, Grimaldi A (2000). Recent techniques for tracing pathways in the central nervous system of developing and adult mammals. Brain Res. Bull..

[65] Reiner A (2000). Pathway tracing using biotinylated dextran amines. J. Neurosci. Methods.

