# Supplementary material for: Hyperactivity of Rac1-GTPase pathway impairs neuritogenesis of cortical neurons by altering actin dynamics
Source: Sci Rep. 2018 May 8;8:7254. doi: 10.1038/s41598-018-25354-3 (PMC5940682; doi:10.1038/s41598-018-25354-3)
Supplement: Supplementary file 1 — Supplementary Information [file 41598_2018_25354_MOESM1_ESM.pdf]

Hyperactivity of Rac1-GTPase pathway impairs cortical neurons neuritogenesis by altering actin dynamics

Valentina Zamboni, Maria Armentano, Gaia Berto, Elisa Ciraolo, Alessandra Ghigo, Donatella Garzotto, Alessandro Umbach, Ferdinando DiCunto, Elena Parmigiani, Marina Boido, Alessandro Vercelli, Nadia El-Assawy, Alessandro Mauro, Lorenzo Priano, Luisa Ponzoni, Luca Murru, Maria Passafaro, Emilio Hirsch and Giorgio R. Merlo

Supplementary Figures

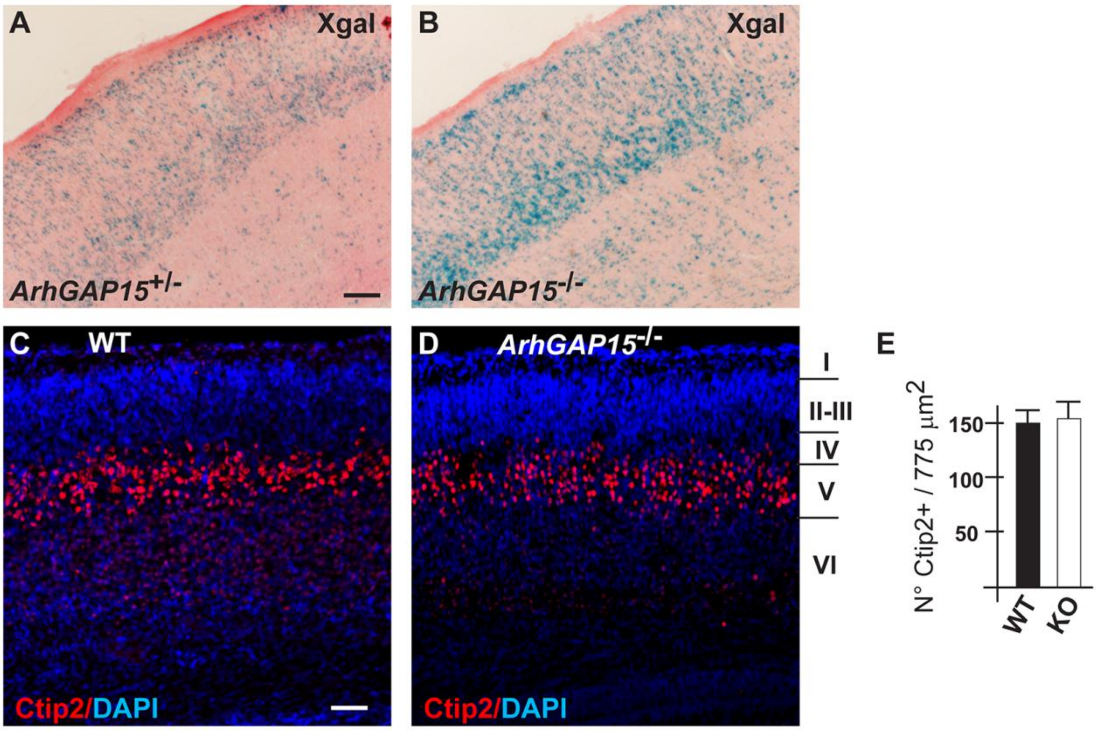

Supplementary Figure S1. Number and distribution of ArhGAP15+ and Ctip2+ neurons in the absence of ArhGAP15

(A,B) Xgal staining of coronal sections of the somatosensory cortex from *ArhGAP15*$^{+/-}$ (A) and from *ArhGAP15*$^{-/-}$ (B) brain, at the age P60. Note the increased signal intensity but the equal distribution of Xgal+ cells. Scale bar in A=50 μm

(C,D) Immunostaining of the somatosensory cortex of WT (C) and *ArhGAP15*$^{-/-}$ (D) brains, at the age P1, with anti-Ctip2 antibody (red nuclear fluorescence), counterstained with DAPI. Layers are indicated on the right of panel D. At this age, staining is observed only in layer V. Scale bar in C=50 μm.

(E) Histogram of the quantification of the number of Ctip2+ nuclei/area in the two genotypes. No significant difference is observed.

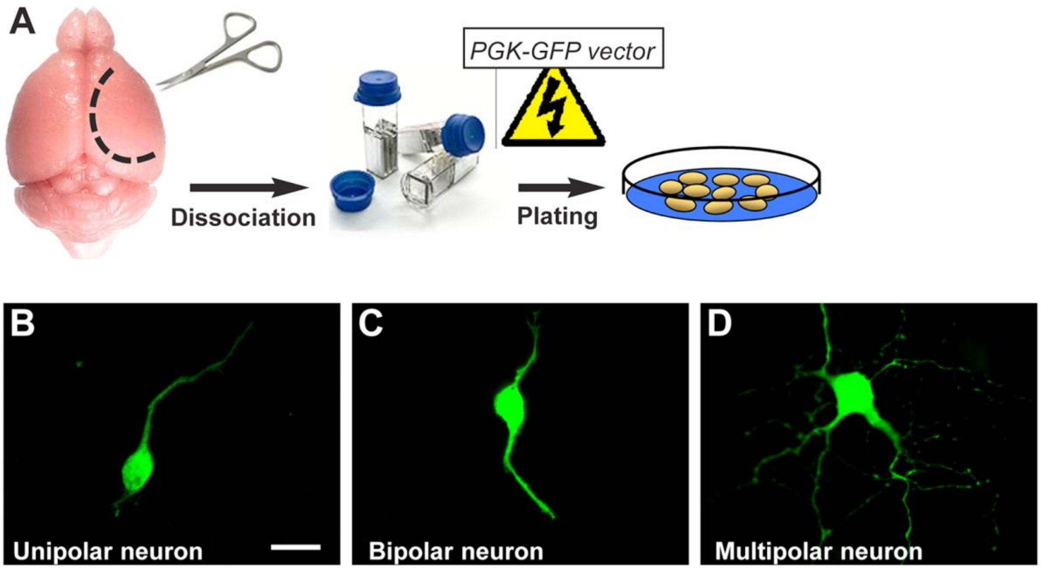

Supplementary Figure S2. Analysis of neuronal morphology of cultured cortical neurons

(A) Illustration of the experimental scheme. Dissociated cells were obtained from from WT or *ArhGAP15*$^{-/-}$ brains at the age E15.5. Cells were electroporated with a PGF-GFP expression vector prior to plating, and examined after 3 DIV.

(B-D) Representative micrographs of GFP-labelled neurons showing, respectively, one (unipolar, in B), two (bipolar, in C) or > two (multipolar, in D) neurites. Scale bar in B=20 μm.

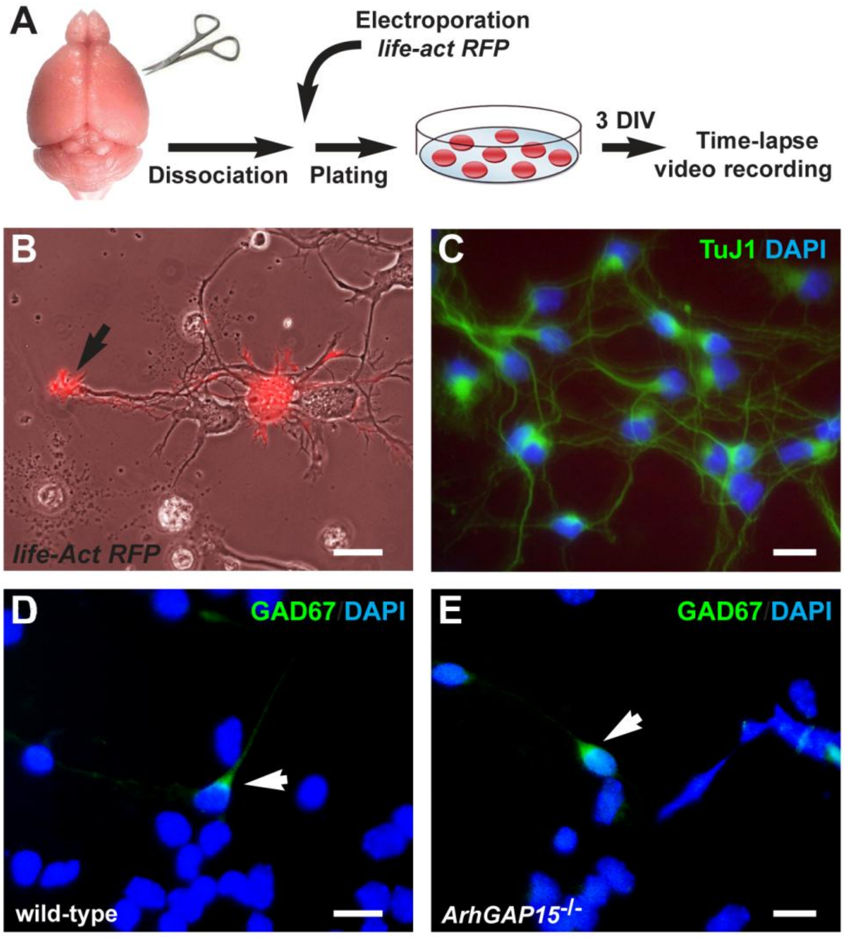

Supplementary Figure S3. Characterization of cultured cortical neurons, used for Time-Lapse video recording

(A) Scheme illustrating the general experimental strategy. Neurons from dissociated E15.5 cortices were electroporated with LifeAct-RFP (red fluorescence) vectors, then plated for 2 days. At least 50 RFP+ growth cones per genotype were subjected to time-lapse video recording.

(B) Representative micrograph of electroporated neurons transfected with life-act-RFP (red fluorescence), cultured from dissociated cortices. Black arrow indicates a RFP+ growth cone.

(C) Micrograph of immunostaining to detect βIII-tubulin (TuJ1 antibody, green fluorescence) on 2 DIV cultures of cortical neurons. Nuclei are counterstained with DAPI.

(D,E) Micrographs of immunostaining to detect GAD67 (green fluorescence, white arrowheads) on cultures of wild-type (D) or *ArhGAP15*$^{-/-}$ (E) cortical neurons, to verify the proportion of GABAergic neurons. Nuclei are counterstained with DAPI. GAD67+ neurons are present in equal proportions (8%) in both genotypes. Scale bars in all panels=10 μm.

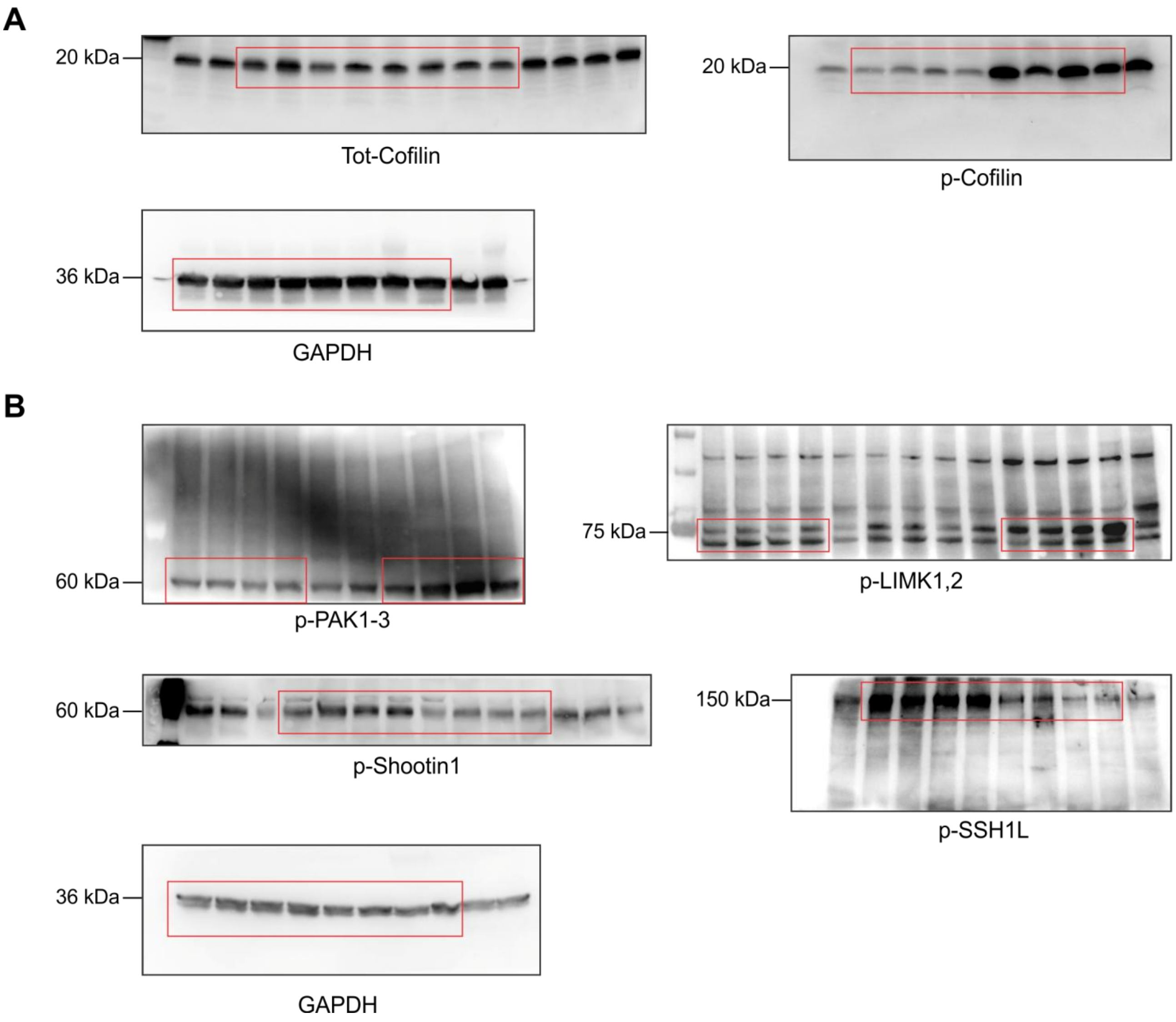

Supplementary Figure S4. Full-length blots corresponding to Fig. 5

(A) Indicated parts (red box) are shown in Fig. 5C.
(B) Indicated parts (red box) are shown in Fig. 5D.
